# Paradoxical Topological Soliton Lattice in Anisotropic Frustrated Chiral Magnets

**DOI:** 10.1002/advs.202514568

**Published:** 2025-11-06

**Authors:** Sayan Banik, Nikolai S. Kiselev, Ashis K. Nandy

**Affiliations:** ^1^ School of Physical Sciences National Institute of Science Education Research Jatni 752050 India; ^2^ Homi Bhabha National Institute Training School Complex, Anushakti Nagar Mumbai 400094 India; ^3^ Peter Grünberg Institute Forschungszentrum Jülich 52425 Jülich Germany

**Keywords:** anisotropic, chiral, frustration, skyrmion‐antiskyrmion lattice, topological magnet

## Abstract

2D chiral magnets are known to host a variety of skyrmions, characterized by an integer topological charge (Q∈Z). However, these systems typically favor uniform lattices as thermodynamically stable phases composed of either skyrmions (*Q* = −1) or antiskyrmions (*Q* = 1). In isotropic chiral magnets, skyrmion‐antiskyrmion coexistence is typically transient due to mutual annihilation, making the observation of a stable, long‐range, ordered lattice a significant challenge. Here, this challenge is addressed by demonstrating a skyrmion‐antiskyrmion lattice as a magnetic field‐induced topological ground state in chiral magnets with competing anisotropic interactions, specifically Dzyaloshinskii‐Moriya and frustrated exchange interactions. This unique lattice exhibits a net‐zero global topological charge due to the balanced populations of skyrmions and antiskyrmions. Furthermore, density functional theory and spin‐lattice simulations identify 2Fe/InSb(110) as an ideal candidate material for realizing this phase. This finding reveals new possibilities for manipulating magnetic solitons and establishes anisotropic frustrated chiral magnets as a promising material class for future spintronic applications.

## Graphical abstract

The article describes the discovery of a stable skyrmion‐antiskyrmion lattice (S‐AL) in anisotropic frustrated chiral magnets. This lattice has a net‐zero topological charge due to a balanced population of skyrmions and antiskyrmions. This is a paradoxical finding since these particles normally annihilate each other. The S‐AL phase emerges as a magnetic field induced ground state, revealing new possibilities for manipulating magnetic solitons.

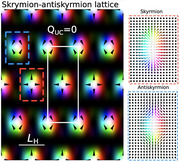

## 1 Introduction

Topological magnetic solitons^[^ ^1^ ^]^ are magnetization field configurations that maintain stable shapes and sizes over time and cannot be continuously transformed into trivial configurations, such as a saturated ferromagnet (FM). They can also move and interact with each other like ordinary particles. Chiral magnets are the most prominent example of a magnetic system exhibiting a wide diversity of topological magnetic solitons.^[^ ^2^, ^3^, ^4^, ^5^, ^6^, ^7^, ^8^, ^9^, ^10^, ^11^, ^12^, ^13^, ^14^ ^]^ We commonly refer to these solitons as chiral magnetic skyrmions. In these systems, magnetic skyrmions are stabilized by the competition between Heisenberg exchange interaction and chiral Dzyaloshinskii‐Moriya^[^ ^15^, ^16^ ^]^ interaction (DMI). The existence of statically stable chiral magnetic skyrmions was first predicted theoretically^[^ ^17^ ^]^ and later confirmed experimentally through both direct^[^ ^18^, ^19^, ^20^, ^21^, ^22^, ^23^, ^24^, ^25^ ^]^ and indirect^[^ ^26^ ^]^ observations of their hexagonal lattice arrangement in various compounds.

The diversity of magnetic skyrmions can be classified using the homotopy group concept. In the case of 2D, the classifying group is the second homotopy group with respect to the $S^{2}$ sphere, which is known to be isomorphic to the group of integers, $\pi_{2}\left(S^{2}\right)=Z$. The latter means that each topological soliton can be associated with a specific integer called the skyrmion topological charge:

(1) $$Q=\frac{1}{4\pi }\int_{\Omega }m\cdot \left[\partial_{x}m\times \partial_{y}m\right]\;dx\;dy$$

where **m**(*x*, *y*) = **M**/|**M**| is the unit vector field of magnetization, and Ω is the skyrmion localization area chosen such that at its boundary ∂Ω, the magnetization field points in one direction, **m**(∂Ω) = **m**_0_. Following the standard convention for sign definiteness in Equation (1), we assume a right‐handed Cartesian coordinate system and that magnetization at the boundary satisfies the criterion, **m**_0_ · **e**_*z*_ > 0. For details, see Refs. [2, 27].

The solitons of opposite topological charges are typically called skyrmions (*Q* = −1) and antiskyrmions (*Q* = 1). In isotropic systems, depending on the crystallographic symmetry, only one type of particle, either skyrmion or antiskyrmion, is energetically most favorable. These particles form periodic lattices with long‐range order in a specific range of the external magnetic field. Such lattices, composed of one sort of particle, either skyrmions, or antiskyrmions, have been experimentally observed in various compounds.^[^ ^18^, ^19^, ^20^, ^21^, ^22^, ^23^, ^24^, ^25^, ^26^, ^28^ ^]^

In this work, we report the paradoxical phenomenon of a stable regular lattice composed of both skyrmions and antiskyrmions. The paradox stems from the fact that, under normal conditions, skyrmion, and antiskyrmion act as particle‐antiparticle pairs, exhibiting mutual annihilation or spontaneous generation phenomena,^[^ ^27^, ^29^ ^]^ much like an electron and a positron. We demonstrate that in systems with anisotropic interactions, including DMI and frustrated exchange, the skyrmion‐antiskyrmion lattice (S‐AL) carrying net‐zero topological charge has an equilibrium period and becomes the lowest energy state within a specific range of external magnetic fields. Our claims are supported by an anisotropic micromagnetic model, followed by density functional theory (DFT) calculations for a realistic system and corresponding spin lattice model analyses. In particular, we demonstrate a prototype 2D chiral magnet in which the discussed phenomenon occurs. This discovery represents a vital element of the general theory of topological solitons in chiral magnetic systems and beyond.

The paper is organized as follows. First, we introduce the minimal micromagnetic model that predicts the S‐AL phase as the ground state in 2D magnetic systems–a class of magnets we term anisotropic frustrated chiral magnets. We discuss the connection between our model and other systems previously studied in the literature, highlighting the role of specific model parameters in stabilizing the S‐AL. Additionally, we report the formation of stable clusters composed of equal and unequal numbers of skyrmions and antiskyrmions, demonstrating that annihilation is not a necessary outcome. Following this, we present a realistic heterostructure–a Fe double layer on an InSb(110) semiconductor substrate–where our DFT calculations and analysis of the corresponding spin lattice model predict the S‐AL phase as the energetically favorable lowest energy state in a wide range of applied magnetic field.

## 2 2D Magnet with Anisotropic Interactions

We consider the model of 2D magnets, which is composed of Zeeman energy term, frustrated exchange interaction, and DMI. The micromagnetic energy functional can be written as follows:

(2) $$E=\int \left\{E_{z}\left(m\right)+E_{e}\left(m\right)+E_{d}\left(m\right)\right\}t\;dx\;dy$$

where magnetization field **m** ≡ **m**(*x*, *y*) is assumed to be homogeneous along the slab thickness *t*. In the Zeeman energy term, $E_{z}=-\left|M\right|m\cdot B_{\mathrm{ext}}$, the external field is always assumed to be perpendicular to the slab. $E_{e}$ and $E_{d}$ stand for the energy density of Heisenberg exchange interaction and DMI, respectively.

**Figure 1:**
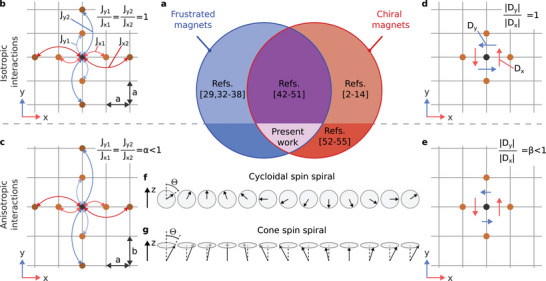
Diversity of systems with frustrated exchange and chiral interactions. a) The diagram illustrates the diversity of systems with frustrated Heisenberg exchange interactions and DMI, including both isotropic and anisotropic cases. The case studied in the present work corresponds to the system with anisotropic interaction parameters, *i*.*e*., the anisotropic frustrated chiral magnet. b) The schematic representation of the minimal model for the 2D magnet with isotropic frustrated exchange interactions on a square lattice where nearest and the next after‐nearest neighbor exchange constants have opposite sign, *J*_1_ > 0 (ferromagnetic) and *J*_2_ < 0 (antiferromagnetic), see the spin‐lattice Hamiltonian Equation (7) in Experimental Section. c) The schematic representation of a rectangular lattice with anisotropic frustrated exchange interactions, characterized by unequal exchange coupling constants along the *x*‐ and *y*‐directions. d) The DMI vectors between the nearest neighbors on a square lattice for the interfacial type DMI. For an isotropic system, the absolute values of the DMI vectors in both *x* and *y* directions are identical. e) The system with anisotropic DMI on a rectangular lattice, where the magnitude of the DMI differs along the *x*‐ and *y*‐directions. f) A cycloidal spin spiral, often resulting from interfacial DMI in chiral magnets, characterized by a varying polar angle Θ with respect to the *z*‐axis along the propagation direction. g) A cone spin spiral, characterized by a fixed polar angle Θ and a varying azimuthal angle along the propagation direction.

It is important to note that considering only anisotropic DMI is often insufficient for accurately describing real systems. A comprehensive and consistent model should account for both exchange and DMI being anisotropic, reflecting the system's inherent symmetry breaking, as shown in **Figure** **1**c,e. Because of that, in our model, the Heisenberg exchange interaction energy with second‐order and fourth‐order terms is represented by

(3) $$E_{e}\;=\;A_{x}\;{\left(\;\frac{\partial m}{\partial x}\;\right)}^{\;\;2}\;+A_{y}\;{\left(\;\frac{\partial m}{\partial y}\;\right)}^{\;\;2}\;+B_{x}\;{\left(\;\frac{\partial^{2}m}{\partial x^{2}}\;\right)}^{\;\;2}\;+B_{y}\;{\left(\;\frac{\partial^{2}m}{\partial y^{2}}\;\right)}^{\;\;2}$$

This energy term represents one of the limiting cases of a more general model,^[^ ^30^ ^]^ describing frustrated magnets. In terms of the atomistic spin‐lattice model, the exchange energy term can be approximated by the Heisenberg exchange between the nearest and the next after‐nearest neighbors, as depicted in Figure 1b,c. The corresponding micromagnetic terms can be obtained by Taylor expansion of the spin‐lattice Hamiltonian with respect to the lattice parameter.^[^ ^30^ ^]^ The micromagnetic terms and spin‐lattice coupling constants in a square lattice are related as follows: $A=(J_{1}/2+2J_{2})/a$ and $B=-(J_{1}/96+J_{2}/6)a$, where *a* is the lattice constant. More details can be found in Note S1 (Supporting Information).^[^ ^31^ ^]^

In the isotropic case, where $A_{x}=A_{y}=A>0$ and $B_{x}=B_{y}=B>0$, the minimum energy of the term Equation (3) corresponds to a collinear FM state. In the case of frustrated exchange interaction, when A<0 and B>0, the ground state of the system is a spin spiral (SS) that is degenerate with respect to the rotation of magnetization **m** about any arbitrary axis. The equilibrium period of such a flat SS is defined by the ratio between exchange coupling constants, $L_{H}=4\pi \sqrt{B/\left|A\right|}$. Under the external magnetic field, its degeneracy with respect to arbitrary rotational axis is broken. In a range of external magnetic fields, 0 ⩽ *B*_ext_ < *B*_c_, the conical SS (cone‐SS) is the lowest energy state. So, there exists a critical field, $B_{c}=A^{2}/\left(4M_{s}B\right)$, above which the cone‐SS phase continuously converges to a saturated FM state. Notably, the period *L*_H_ of the cone‐SS does not depend on the strength of the applied magnetic field. The exchange term Equation (3) can also be seen as a limiting case of the interaction studied in Refs. [32, 33]. In these seminal works, the stability of magnetic skyrmions, driven by higher‐order exchange interactions–what is now often referred to as exchange frustration–was first predicted. Over the years, the concept of skyrmions stabilized by exchange frustration has evolved gradually.^[^ ^29^, ^34^, ^35^, ^36^, ^37^, ^38^ ^]^ However, this type of skyrmions has not garnered as much attention as DMI‐stabilized skyrmions in pure chiral magnets.

In the model Equation (2), we consider the interfacial type DMI, which, in the micromagnetic limit, can be written as

(4) $$E_{d}=D_{x}\Lambda_{xz}^{\left(x\right)}+D_{y}\Lambda_{yz}^{\left(y\right)}$$

where Lifshitz invariants are defined as follows,

(5) $$\Lambda_{ij}^{\left(k\right)}=m_{i}\frac{\partial m_{j}}{\partial k}-m_{j}\frac{\partial m_{i}}{\partial k}$$

In the atomistic spin‐lattice model, it corresponds to the case where the DMI vector, **D**, is perpendicular to the **r**‐vector between the interacting spins, as depicted in Figure 1d,e. Such orientations of the DMI vectors are common in all 2D systems with *C*_*nv*_ symmetry. The micromagnetic DMI constants in the case of a square lattice can be expressed in terms of the spin‐lattice model parameters as follows: $D_{x}=aD_{x}$ and $D_{y}=aD_{y}$. The full range of systems described by model Equation (2) is illustrated in the diagram shown in Figure 1a. Here, this diagram visually distinguishes between three distinct magnetic regimes: pure frustrated magnets (blue), pure chiral magnets (red), and systems with competing exchange frustration and DMI (magenta).

Our model, incorporating interfacial‐type DMI and exchange frustration, supports two distinct types of SSs. When DMI dominates, the system stabilizes into a cycloidal‐SS, as shown in Figure 1f. In the presence of a nonzero magnetic field and dominating exchange frustration, the cone‐SS depicted in Figure 1g emerges as the lowest energy state. These SSs can be characterized by two fundamental properties: chirality, defined as ∼**q** · (∇ × **m**), and polarity, given by ∼|**q** × (∇ × **m**)|, where **q** denotes the SS wave vector. The cycloidal‐SS in Figure 1f exhibits zero chirality but nonzero polarity. In contrast, the cone‐SS in Figure 1g shows both nonzero chirality and polarity. For reference, helical SSs, characteristic of systems with bulk‐type DMI and not shown here, display nonzero chirality but zero polarity.

**Figure 2:**
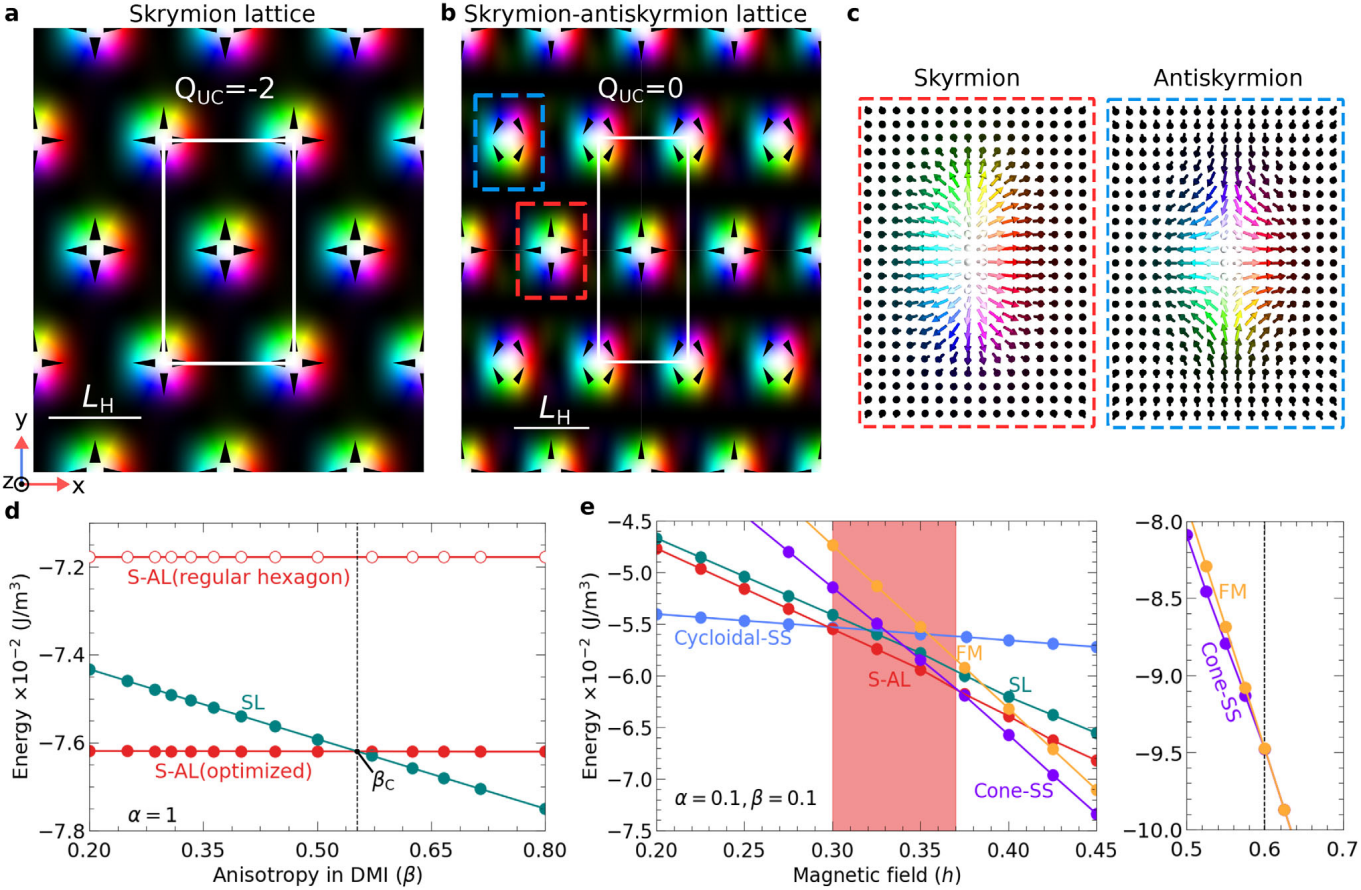
Achieving S‐AL ground states within model Equation (2) using anisotropic parameters: Through micromagnetic simulations utilizing direct energy minimization of our model, we obtain optimized unit cells (white rectangular boxes) for both SL and S‐AL phases, as presented in (a) and (b), respectively. The optimization scheme is detailed in Figure S9 (Supporting Information).^[^ ^31^ ^]^ The magnetization vector field is visualized by standard color code. Here, *Q*_UC_ = 0 signifies the rectangular unit cell which represents the net‐zero topological charge lattice. c) Left and right spin textures depict individual skyrmion and antiskyrmion, respectively, obtained from the optimized S‐AL configuration in (b). d) Energy profiles for the SL and S‐AL phases as a function of β. The S‐AL phase is energetically favored over the SL phase for β < β_c_~(≈ 0.55). The energy curves of the SL and S‐AL phases intersect at the critical value β_c_. The significant energy gain observed in the S‐AL phase through shape optimization (θ optimization) is the primary reason for the high value of β_c_. All calculations are performed under a constant external magnetic field of *h* = 0.35. e) Energies for all competing non‐collinear states–cycloidal‐SS, cone‐SS, SL, and S‐AL–as well as saturated FM state are plotted as a function of *h* for anisotropic frustrated chiral magnets. Here, we fix both α and β to 0.1. The S‐AL phase is identified as the ground state within the red‐shaded regions, corresponding to the lowest energy states for specific ranges of *h*. For a comparison of the system's behavior at higher anisotropy, β > β_c_, see Figure S10 (Supporting Information).^[^ ^31^ ^]^

The key parameters of our model define the anisotropy in the exchange interaction and DMI. The parameter $\alpha =A_{y}/A_{x}=B_{y}/B_{x}$ defines the anisotropy in exchange interaction. In a more general approach, one could consider the case when $\frac{A_{y}}{A_{x}}\neq \frac{B_{y}}{B_{x}}$, but here we ignore this option for simplicity of the model. The anisotropy in DMI is defined by parameter $\beta =D_{y}/D_{x}$. Note that β depends only on the absolute values of the DMI vectors, while their directions are fixed by the lattice symmetry [see Figure 1d and e]. In the following, we show that anisotropic DMI, β < 1, is the key ingredient for stabilization of the S‐AL depicted in **Figure** **2**b.

The parameters α and β can be freely adjusted within the range 0 ⩽ α ⩽ ∞ and 0 ⩽ β ⩽ ∞. However, for definiteness, here, we assume that α ∈ [0, 1] and β ∈ [0, 1]. Therefore, the coupling strengths along the *y*‐axis are always weaker than those along the *x*‐axis. Hereafter, the inherent anisotropy, with the *x*‐axis as the strong axis and the *y*‐axis as the weak axis, gives rise to a distinct class of chiral magnets, specifically designated as anisotropic frustrated chiral magnets. The elongated shapes of skyrmions and antiskyrmions, clearly visible in Figure 2c, are a direct consequence of the anisotropic properties of the exchange interaction and DMI in our model.

In the isotropic case with α = 1, β = 1, and positive exchange coupling constants (A>0 and B≥0), model (2) simplifies to the extensively studied model of isotropic chiral magnets.^[^ ^2^, ^3^, ^4^, ^5^, ^6^, ^7^, ^8^, ^11^, ^17^, ^39^, ^40^, ^41^ ^]^ To date, only systems with isotropic frustrated exchange and isotropic DMI (α = β = 1) have been explored in the literature.^[^ ^42^, ^43^, ^44^, ^45^, ^46^, ^47^, ^48^, ^49^, ^50^, ^51^ ^]^ These systems are represented by the dark magenta region in Figure 1a. Most of the parameter space within the isotropic regime can be attributed to known limiting cases of model Equation (2), which we systematically examine in Note S2 (Supporting Information).^[^ ^31^ ^]^

In contrast, anisotropic systems have been primarily studied in the context of DMI only.^[^ ^52^, ^53^, ^54^, ^55^ ^]^ This includes the limiting case of strongly anisotropic systems corresponding to so‐called monoaxial chiral magnets.^[^ ^54^, ^55^, ^56^ ^]^ The models featuring both anisotropic DMI and anisotropic exchange, as considered in this work with terms Equations (3) and (4), remain largely unexplored. Finally, it is important to emphasize that our model does not include magnetocrystalline anisotropy or any other form of spin orientation anisotropy, as they are not essential for the phenomena discussed here. However, to ensure a comprehensive analysis, we examine the role of magnetocrystalline anisotropy in stabilizing the S‐AL phase, including a 2D material example: the 2Fe/InSb(110) heterostructure.

## 3 Model Parameters

Below, we consider the solutions of the model Equation (2) with $A_{(x)y}<0$ and $B_{x(y)}>0$, assuming for definiteness that $D_{x(y)}>0$. To facilitate further analysis, we introduce two parameters, *L*_D_ and *L*_H_, representing the equilibrium period of the spiral state in two limiting cases. When A→0, the equilibrium period of chiral spiral state is $L_{D}=2\pi \sqrt[{3}]{16B_{x}/D_{x}}$, and when D→0, the exchange frustration driven spiral has period $L_{H}=4\pi \sqrt{B_{x}/\left|A_{x}\right|}$ (see Note S1, Supporting Information^[^ ^31^ ^]^). In the first approximation, the ratio between *L*_H_ and *L*_D_ characterizes the relative contributions of the frustrated exchange and DMI terms to the stability of SS and other noncollinear phases. Frustrated exchange dominates DMI when *L*_H_ < *L*_D_ and vice versa. The reduced magnetic field, *h* = *B*_ext_/*B*_c_, is provided in units relative to the critical field, $B_{c}=A_{x}^{2}/\left(4M_{s}B_{x}\right)$.

Following the standard approach, we perform energy minimization of the functional Equation (2) for various spin configurations with optimized parameters across different values of the external magnetic field. For example, the SS state is optimized with respect to its period and propagation direction. In contrast, various lattices of magnetic skyrmions are optimized in terms of the shape and size of their unit cells. We then compare the energy densities of all phases to determine the lowest energy state as a function of external magnetic fields. The energy minimization was performed using the mumax code^[^ ^57^ ^]^ (see Experimental Section), and the corresponding script is provided in the Supporting Information.

## 4 Results and Discussion

### 4.1 Micromagnetic Model for S‐AL in Frustrated Chiral Magnets

To begin, we illustrate the results of the energy minimization for the case of isotropic exchange, α = 1, but anisotropic DMI, β < 1. In these calculations we keep fixed value of $A_{x}=A_{y}=-10^{-17}$ Jm^−1^, while other parameters ($B_{x}$, $B_{y}$, $D_{x}$, $D_{y}$) are defined by parameter β and fixed values of *L*_H_ = 50 nm, and *L*_D_ = 100 nm (*L*_H_/*L*_D_ = 0.5), see also Note S1 (Supporting Information).^[^ ^31^ ^]^ Figure 2a,b depict the equilibrium SL and S‐AL configurations, respectively. As detailed in Figure S9 (Supporting Information),^[^ ^31^ ^]^ the equilibrium lattice phase corresponds to the energy minimum in the parameter space defined by the dimensions of the rectangular unit cell in both directions. The equilibrium SL and S‐AL configurations shown in Figure 2a,b correspond to a magnetic field of *h* = 0.35 applied along the negative *z*‐axis. The S‐AL phase exhibits a balanced population of *Q* = ±1 topological charges, forming a lattice with net‐zero topological charge (*Q*_UC_ = 0) per unit cell. In this phase, skyrmions and antiskyrmions elongate (Figure 2c), causing noticeable distortion from the ideal hexagonal lattice. In contrast, the skyrmion elongation in the SL phase at β = 0.1 is minimal, and the resulting lattice distortion is not noticeable. Moreover, the equilibrium unit cell of S‐AL is slightly larger than that of SL. As illustrated in Figure 2d, when both the SL and the S‐AL phases are constrained to a regular hexagonal symmetry and optimized solely with respect to the scaling parameter, the energy of the S‐AL phase increases significantly. The scaling parameter refers to the uniform scaling of the rectangular unit cell in both directions. This emphasizes the necessity of optimizing both lattice parameters to achieve the lowest‐energy lattice shape.

Most importantly, Figure 2d shows that with varying anisotropy in the DMI, the energy of S‐AL can become lower than that of SL. In this case, the critical value of the anisotropy parameter at which the energies of SL and S‐AL are equal is around β_c_ ≈ 0.55, while, strictly speaking, β_c_ is a function of *h*. This is detailed in Figure S11d (Supporting Information).^[^ ^31^ ^]^ We conclude that anisotropic DMI can reverse the energy balance between SL and S‐AL. Anisotropic DMI alone does not make the S‐AL phase ground state. In the case of isotropic exchange (α = 1), both SL and S‐AL remain metastable, meaning their energies are higher than those of other phases. Thus, while anisotropic DMI is necessary in our model, it is not sufficient to make S‐AL the lowest energy state within a specific field range.

In the case of anisotropic interactions, the energy dependencies of the phases differ. For simplicity, but without loss of generality, we consider the case of α = β. In Figure 2e, we present the energies of the various magnetic phases as a function of applied magnetic field, calculated for the anisotropic frustrated chiral magnets with α = β = 0.1. At low field strengths, the cycloidal‐SS phase is the ground state, and it undergoes a first‐order phase transition to the S‐AL phase at a critical field of *h* ≈ 0.3. A subsequent first‐order phase transition occurs at *h* ≈ 0.37, beyond which the cone‐SS phase emerges as the ground state. Consequently, the S‐AL phase is stabilized within a well‐defined range of magnetic fields, emphasizing the crucial role of anisotropic interactions in determining the ground state. Noticeably, the ordinary SL remains a metastable state throughout the entire field range, with its energy curve nearly parallel to that of the S‐AL phase. As the magnetic field is further increased, a second‐order phase transition takes place at approximately *h* ≈ 0.6, leading to a transition from the cone‐SS to the saturated FM state (as indicated by the vertical line in the rightmost panel of Figure 2e.) To gain deeper insights into the influence of anisotropic exchange interactions on the stability of the S‐AL phase, we present additional analyses in Figures S10 and S11 (Supporting Information).^[^ ^31^ ^]^ As in Figure S12 (Supporting Information),^[^ ^31^ ^]^ spin texture elongation within the S‐AL phase is a direct consequence of the system seeking the lowest energy state. Within the constraints of our chosen DMI configuration, elongation along the *y*‐direction is energetically most favorable with a deformed hexagonal lattice.

We conclude that the S‐AL phase emerges as the lowest energy state within a specific magnetic field range only when β < β_c_, and the anisotropy parameter α is below a critical value. In the case of Figure 2e, for β = 0.1, the critical value of anisotropy in exchange interactions α is found to be ≈0.26. Reducing α below this critical value favors the S‐AL phase as the ground state in a broader window of applied fields.

It is worth noting that, in general, β_c_ is a function of the model parameters. As shown in Figure S11c (Supporting Information),^[^ ^31^ ^]^ the dependence of the critical anisotropy parameter β_c_ on the *L*_H_/*L*_D_ ratio for different values of α, with *h* held constant at 0.35. Notably, for any given value of α, there exists a finite range of *L*_H_/*L*_D_ where 0 < β_c_ < 1.

**Figure 3:**
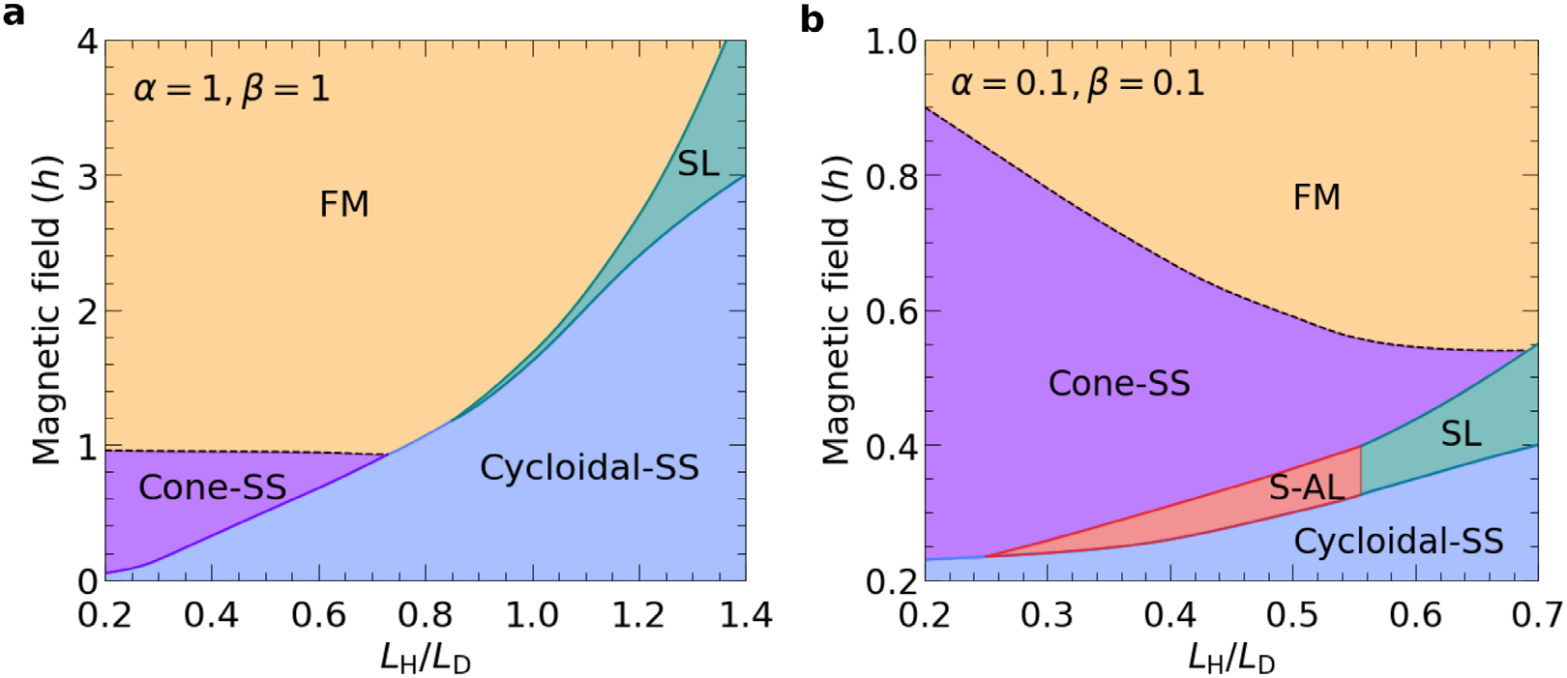
The phase diagrams in the *L*_H_/*L*_D_‐*h* plane. a) The phase diagram for the isotropic magnets. The parameter space is clearly divided into two regions: one dominated by exchange frustration and the other by DMI. Decreasing the ratio *L*_H_/*L*_D_ (enhancing exchange frustration) stabilizes the cycloidal‐SS phase in the absence of external magnetic field *h*. Further, under an external field *h*, a cone‐SS phase appears, with SL and S‐AL as metastable states. In this region, the system exhibits three energetically favored phases: cycloidal‐SS, cone‐SS, and FM. The dashed line between cone‐SS and FM denotes the second‐order phase transition between them. Dominant DMI interactions are evidenced by the expansion of the cycloidal‐SS ground state region at higher *L*_H_/*L*_D_ ratios. Typically, the cone‐SS phase disappears under an external field in the strong DMI limit, while the cycloidal‐SS phase undergoes a first‐order phase transition to the SL phase. b) The phase diagram for the anisotropic frustrated chiral magnet. At *L*_H_/*L*_D_ ≈ 0.56, the SL and S‐AL phases exhibit equal energy across a range of *h*, defining a boundary between these two distinct lattice phases. The SL phase becomes energetically favorable compared to the S‐AL phase upon increasing further *L*_H_/*L*_D_ ratios. This can be attributed to the fact that the critical value of β_*c*_ remains below 0.1 in this regime.

In addition to the anisotropy parameters α and β, a key parameter in the model is the ratio *L*_H_/*L*_D_. The emergence of the S‐AL as the lowest energy phase within a specific range of applied magnetic fields requires particular values of all three parameters. This is illustrated by the magnetic phase diagrams shown in **Figure** **3**.

First, let us consider the isotropic case (α = β = 1) where S‐AL phase does not emerge as a stable phase, Figure 3a. For low values of *L*_H_/*L*_D_, the system exhibits typical behavior of 2D exchange‐frustrated magnets.^[^ ^36^ ^]^ In particular, as *L*_H_/*L*_D_ → 0, the cone‐SS phase becomes the lowest energy state across the entire field range, from zero up to saturation at *h* = 1. With increasing DMI contributions (increasing *L*_H_/*L*_D_), the conical phase starts to compete with the cycloidal‐SS. For *L*_H_/*L*_D_ ≳ 0.7, the stability range of the cycloidal‐SS phase extends beyond the saturation field of the cone‐SS, *h* > 1, effectively eliminating the conical phase. As the ratio increases further to *L*_H_/*L*_D_ > 0.85, in the applied field, the system exhibits a first‐order phase transition from a cycloidal‐SS to a hexagonal SL, followed by another first‐order transition to a saturated FM state. This behavior is typical of isotropic chiral magnets, where DMI competes with exchange frustration.^[^ ^45^ ^]^ Notably, the transition from the SL phase to the saturated state in such frustrated chiral magnets represents a first‐order phase transition. In systems with dominating DMI, isolated antiskyrmions remain stable only in a narrow range of fields.^[^ ^4^ ^]^ In these conditions, the S‐AL phase obviously cannot be stable.

Figure 3b shows the phase diagram for the case of anisotropic exchange interactions and DMI, with α = β = 0.1. In this scenario, the S‐AL phase appears within the intermediate range of 0.24 ⩽ *L*_H_/*L*_D_ ⩽ 0.56. Similar to the isotropic model discussed above, the lower and upper bounds of this range can be attributed to the effective transition of the system to the models of anisotropic exchange‐frustrated magnet and anisotropic chiral magnet,^[^ ^52^, ^55^ ^]^ respectively. In the pure model of a chiral magnet–without exchange‐frustration–even at relatively weak anisotropy in DMI, β ≲ 0.7, the SL becomes unstable.^[^ ^58^ ^]^ In our model, for *L*_H_/*L*_D_ ≳ 0.56, the SL remains stable even for β = 0.1 exclusively due to the presence of exchange frustration.

The emergence of the S‐AL phase as the lowest energy state can be explained by its distinct magnetization properties. The S‐AL phase is characterized by magnetization modulations with the opposite helicity angle,

(6) $$\gamma =\text{arctan2}\;\left(m\cdot \hat{\varphi },\;m\cdot \hat{\rho }\right)$$

where $\hat{\rho }$ and $\hat{\varphi }$ denote the radial and azimuthal unit vectors in the film plane, respectively, along orthogonal directions (see Figure 2c). When the DMI coupling strength is reduced along the weak axis (*y*‐direction), the total DMI energy contribution remains nearly unchanged. This occurs because along the *y*‐axis, the helisity angle of cycloidal modulations in the skyrmion chain is γ = 0 while along the chain of antiskyrmions γ = π. These chains of skyrmion and antiskyrmions are well seen in Figure 2b. As a result, the DMI contribution to the energy of the S‐AL phase tends to be independent of the DMI strength along the *y*‐axis and is thus independent of β. In contrast, the DMI contribution to the energy of the SL phase is highly sensitive to DMI anisotropy. As β decreases, the energy of the SL phase rises, while the energy of the S‐AL phase remains unaffected. It is important to note that this mechanism is feasible only in the presence of sufficiently strong exchange frustration. In systems where DMI dominates, the S‐AL phase is inherently unstable. Conversely, in systems where exchange frustration dominates, the stable phases below the saturation field are limited to cone‐SS and cycloidal‐SS. Our analysis shows that the energy balance can shift in favor of the S‐AL phase by introducing anisotropy (α) into the exchange energy terms.

In conclusion, the stability of the S‐AL phase relies on three critical factors: I) DMI and exchange frustration must contribute comparably to the energy of SS (*L*_H_ and *L*_D_ are of the same order). II) The exchange interaction must exhibit anisotropy α ≠ 1. III) The DMI must also be anisotropic β ≠ 1. Notably, our analysis also indicates that the stability of the S‐AL phase requires correlated anisotropy in exchange and DMI, ensuring that the hard exchange axis coincides with the hard DMI axis (α < 1 and β < 1). This alignment reflects the natural behavior of magnetic systems with broken symmetry, confirming that the micromagnetic model presented here corresponds to a realistic physical system.

To gain a deeper understanding of the factors stabilizing the S‐AL phase, we extended our 2D model to include the magnetocrystalline anisotropy energy term (see Experimental Section for details). As shown in Figure S13 (Supporting Information),^[^ ^31^ ^]^ the easy‐axis uniaxial anisotropy significantly favors the cone‐SS and FM phases over the lattice phase. Consequently, the critical field for the S‐AL to cone‐SS phase transition decreases with increasing uniaxial anisotropy, narrowing the magnetic field window for the S‐AL phase.

While skyrmion‐antiskyrmion pairs generally exhibit a strong tendency towards annihilation, the anisotropic model can stabilize metastable clusters of both even and odd numbers of skyrmions and antiskyrmions under finite magnetic fields. The attractive interaction between skyrmions and antiskyrmions in these clusters is evident from the snapshots provided in Figure S14 (Supporting Information).^[^ ^31^ ^]^ It also demonstrates their stability over a wide range of applied magnetic fields. More importantly, the skyrmions and antiskyrmions are found to form stable configurations without annihilation. At low magnetic fields (*h* = 0.5), the cone‐SS phase represents the global energy minimum, and all clusters in Figure S14a,d (Supporting Information),^[^ ^31^ ^]^ regardless of their composition, remain stable within the conical background magnetization. In the pure model of a frustrated magnet, similar clusters embedded in the cone‐SS, but consisting of a single type of particle, were previously discussed in Ref. [38]. As the magnetic field increases beyond the critical field for the second‐order phase transition to FM state (*h* > 0.7), the background magnetization becomes homogeneous, but all clusters remain intact (see Figure S14e–h, Supporting Information ^[^ ^31^ ^]^).

The above analysis of the micromagnetic model indicates that the criterion for the emergence of S‐AL as the lowest energy state is met over a broad range of model parameters. Therefore, it is reasonable to expect the stable S‐AL phase to appear in real systems. As a proof of concept, in the following section, we present a 2D chiral magnet, identified through DFT calculations and spin‐lattice simulations, as a promising candidate for the experimental observation of the phenomena described above.

## 5 Potential Real 2D Magnet: DFT and Atomistic Spin‐Lattice Simulations

**Figure 4:**
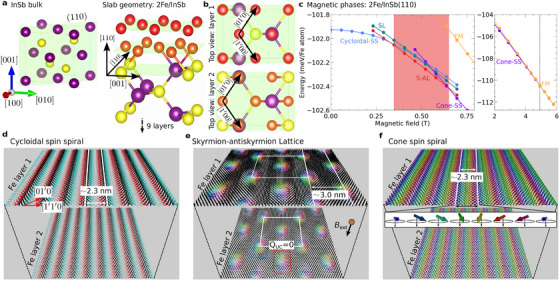
2Fe/InSb(110), a prototypical 2D magnetic heterostructure: a) The slab geometry: a two‐atomic‐layer‐thick Fe film grown on an InSb(110) substrate, forming a magnet/semiconductor heterostructure. b) The thin magnetic layer on the surface of a semiconductor adopts a lattice structure with 2D crystallographic axes, [1′00] and [01′0]. c) Within atomistic lattice model Equation (7), energy lines representing all competing phases are plotted against the external magnetic field *B*_ext_, applied perpendicular to the heterostructure. The S‐AL phase, highlighted by the red region, is the ground state within a specific range of *B*_ext_. The first‐order phase transitions occur at the boundaries of this range: from the cycloidal‐SS to the S‐AL at low fields, and from the S‐AL to the cone‐SS at higher fields. The rightmost panel further confirms the second‐order phase transition between the cone‐SS and FM phases, as evidenced by the merging of energy lines beyond the vertical line. d) Spin configuration of the zero‐field cycloidal‐SS ground state, obtained within spin‐lattice simulations. e) The S‐AL phase at *B*_ext_ = 0.5 Tesla. In this materials, the S‐AL is also a charge (topological) neutral state due to equal number *Q* = ±1 topological charges. Similar to the micromagnetic model, the representing unit cell carries *net‐zero* topological charge *i*.*e*., *Q*_UC_ = 0. f0 Cone‐SS phase at *B*_ext_ = 0.9 Tesla. To improve visibility, the identical magnetic states in both Fe layers are spatially separated.

To demonstrate S‐AL stability in real material, we present a two‐atomic‐layer‐thick Fe film on an InSb(110) substrate, as depicted in **Figure** **4**a,b. It is worth noting that we initially studied the 2Fe/InSb(110) system to explore magnetically ordered 2D structures on semiconducting substrates. Simulations based on DFT‐derived parameters revealed the S‐AL state as the lowest‐energy configuration. This unexpected result prompted the development of a micromagnetic model that explains the stabilization mechanism and generalizes the findings beyond this specific material. The zincblende InSb is a semiconductor that has a notably strong spin‐orbit coupling^[^ ^59^ ^]^ and a well‐characterized (110) surface.^[^ ^60^ ^]^ The surface unit cell is rectangular, and each layer is characterized by a distinct arrangement of In and Sb atoms. The deposition of Fe films on InSb(110) surfaces can induce a significant interfacial DMI among magnetic Fe atoms. This DMI, stemming from SOC and broken inversion symmetry at the interface (Figure 4a), is essential for stabilizing chiral magnetic states, especially in systems where frustrated exchange interactions primarily govern the SS state.

In contrast to traditional 2D chiral magnets, which often utilize heavy‐metal substrates,^[^ ^22^, ^42^, ^45^, ^61^, ^62^ ^]^ our 2D slab geometry integrates a binary semiconductor, leading to a distinctive interfacial configuration. This design is inspired by the well‐characterized 2Fe/GaAs(110) interface,^[^ ^63^, ^64^, ^65^ ^]^ which provides a compelling rationale for our choice of a similar interface. Furthermore, the (110) binary semiconductor surface, with its characteristic *C*_1*v*_ symmetry,^[^ ^66^ ^]^ is essential for providing the anisotropic interaction parameter space as established in our micromagnetic model. In the magnetic unit cell, each of the two magnetic layers (the surface and subsurface layers), consists of four Fe atoms. Each layer in the substrate, however, contains only two atoms, In and Sb. This arrangement yields inequivalent magnetic sites, strikingly different from the standard single‐atom‐per‐layer ultrathin 2D magnets.

We have employed ab initio electronic structure calculations to characterize the relaxed interface of the 2Fe/InSb(110) slab geometry. As shown in Table S1 (Supporting Information), the magnetic moments of the Fe atoms in the 2Fe/InSb(110) sample are consistent with values previously reported for the 2Fe/GaAs(110) system.^[^ ^64^ ^]^ The relaxed magnetic slab is then used to calculate all material‐specific atomistic interaction parameters, including exchange, DMI, and magnetocrystalline anisotropy. For exchange and DMI, calculations extend beyond nearest neighbors to include several neighboring shells around each magnetic atom. This approach allows the parameters in the magnetic layers to capture both intra‐ and interlayer spin‐spin interactions. Importantly, these atomistic parameters exhibit long‐range behavior and display competing signs for each Fe atom. Our first‐principles simulation methodology is detailed in the Methods section, and DFT‐based calculations of magnetic interaction parameters are presented in Note S3 (Supporting Information).^[^ ^31^ ^]^

The magnetic ground state is computed through full parameterization of the spin‐lattice Hamiltonian Equation (7), followed by atomistic spin dynamics simulations performed using the SPIRIT code.^[^ ^67^ ^]^ For computational simplicity, we assume that all Fe atoms possess an identical average magnetic moment of µ_s_ = 2.71~µ_B_, a reasonable approximation. Considering only Heisenberg exchange interactions, the exchange frustration driven magnetic solution is a SS state with a period of approximately 2.9 nm. This is detailed in Note 3 (Supporting Information).^[^ ^31^ ^]^

Upon inclusion of all interaction parameters, our atomistic simulations reveal a left‐handed cycloidal‐SS ground state with a period of 2.3 nm, as shown in Figure 4d. Notably, such atomic‐scale SS states are often observed in ultrathin magnetic films grown on heavy‐metal substrates.^[^ ^20^, ^45^, ^62^ ^]^ In these systems, the interplay of exchange frustration and DMI typically governs the atomic‐scale magnetic texture, with DMI dictating the specific axis of rotation. The presence of DMI often results in a slightly shorter period for the SS compared to that induced by exchange frustration alone. To gain further insights into the system's behavior, we subject it to an external magnetic field, *B*_ext_, oriented perpendicular to the magnetic film. This is modeled by adding a Zeeman energy term, $-\sum_{i}\mu_{s}B_{\mathrm{ext}}\cdot {\hat{m}}_{i}$, to our Hamiltonian Equation (7).

Consistent with our micromagnetic model, we observed magnetic field‐induced phase transitions, as presented in Figure 4c. The cycloidal‐SS state is the lowest energy magnetic configuration below the critical field, *B*_ext_ ≈ 0.35 Tesla. Upon exceeding the critical field, a first‐order phase transition results into a S‐AL phase, as shown in Figure 4e. A subsequent first‐order phase transition transforms the S‐AL phase into a cone‐SS phase (see Figure 4f) at a higher critical field, ≈ 0.65 Tesla. A critical external magnetic field of approximately five Tesla results in a second‐order phase transition to the saturated state. Beyond this point, the energy lines corresponding to the cone‐SS and saturated FM phases coalesce, as shown in the rightmost panel of Figure 4c. Remarkably, the 2Fe/InSb(110) system exhibits the same sequence of phase transitions as predicted by our micromagnetic model. It is also noteworthy that in agreement with the micromagnetic simulations, both skyrmions and antiskyrmions exhibit an elongated shape. These results collectively show that utilizing a semiconductor substrate to lower the symmetry is the crucial factor. This approach not only elucidates the stabilization mechanism but also proves its broad applicability to other material systems.^[^ ^66^ ^]^

Consistent with the micromagnetic model, the ordinary SL phase is also stable in our spin‐lattice model. As illustrated in Figure 4c, this phase exhibits a higher energy compared to the S‐AL phase. The remarkable congruence between Figure 2g (micromagnetic simulations) and Figure 4c (atomistic spin‐lattice simulations), both unequivocally depicting the S‐AL as the ground state, emphasizes the distinctive nature of chiral magnets with anisotropic interactions.

## 6 Conclusion

In conclusion, we have demonstrated a paradoxical phenomenon that has not been reported earlier. Specifically, we have demonstrated that an S‐AL, composed of an equal number of skyrmions and antiskyrmions, becomes the lowest‐energy state of 2D magnets under specific conditions. This phase is characterized by a net‐zero magnetic topological charge. Since skyrmions and antiskyrmions possess opposite topological charges, one would expect them to annihilate. However, we demonstrate that the S‐AL stabilizes by the interplay of frustrated exchange and DMI in systems with interaction axes of both weak and strong character. This defines a distinct class of materials we term anisotropic frustrated chiral magnet. Additionally, our findings indicate that while uniaxial magnetocrystalline anisotropy is not a prerequisite for stabilizing the S‐AL phase, it does favor the cone‐SS and FM phases and consequently reduces the stability range of the S‐AL phase. Moreover, by combining DFT calculations with atomistic spin dynamics simulations, we propose 2Fe/InSb(110) as a realistic heterostructure for the experimental observation of this phenomenon. Our findings show that the S‐AL in 2Fe/InSb(110) remains stable down to the atomic scale. Our micromagnetic simulations reveal a specific mechanism that stabilizes these magnetic phases in low‐symmetry interfacial systems. This mechanism operates particularly well in a geometry like the (110) surface of semiconductors, due to its *C*_1*v*_ symmetry. This work not only presents the discovery of a new magnetic phase but also positions 2D chiral magnets with anisotropic interactions as promising materials for both fundamental research and practical applications.

## 7 Method Section

### Micormagnetic Simulations

The micromagnetic model Equation (2) was investigated using Mumax code.^[^ ^57^ ^]^ Direct energy minimization starting with different initial spin configurations was used to find the equilibrium configurations.

To investigate the role of magnetocrystalline anisotropy, the following energy term was added in Equation (2): $E_{a}=-Kn_{z}^{2}$, where *K* is the anisotropy constant, and the reduced anisotropy parameter is *u* defined by *u* = *K*/(*M*_s_ *B*_c_).^[^ ^68^ ^]^ The sign of *u* defines the easy‐axis (*u* > 0) or easy‐plane (*u* < 0) anisotropy, respectively. Figure S13a,b (Supporting Information)^[^ ^31^ ^]^ illustrate the effect of magnetocrystalline anisotropy on the energy balance in the studied system.

### Equilibrium State Calculation:

In this micromagnetic calculations, the size of the simulated domain is *L*_*x*_ × *L*_*y*_ × 1 nm^3^, along the *x*, *y*, and *z* axes, respectively. To estimate the equilibrium energy density of different phases, direct energy minimization was utilized. To implement the higher‐order exchange energy terms Equation (3), a custom effective field function built was used into Mumax. This approach had been used earlier in Refs. [6, 8]. All calculations had been performed under periodic boundary conditions (PBC).

First, it was determined that the equilibrium propagation direction of the SS aligns with the (**e**_*x*_, **e**_*y*_, 0) vector. To identify the equilibrium period of the SS state, the dimensions of the square domain, *L*_*x*_ = *L*_*y*_ were systematically varied, which consisted of a 128 × 128 × 1 cuboids. The initial configurations were approximated by a homogeneous cycloidal‐SS, with its period *P* chosen to be commensurate with the domain diagonal, satisfying the condition $2P=\sqrt{L_{x}^{2}+L_{y}^{2}}$. For detailed insights into the initial implementation of the SS state, the reader was referred to the Mumax Script I.

The equilibrium SL and S‐AL phases were determined by energy minimization of a rectangular unit cell, where skyrmion cores were strategically positioned at its center and four corners, as illustrated in the inset of Figure S9a,b (Supporting Information).^[^ ^31^ ^]^ This unit cell was embedded within a rectangular simulated domain, whose dimensions *L*_*x*_ and *L*_*y*_ could be tuned to control two key parameters: the core‐to‐core distance $d={\left(L_{x}^{2}+L_{y}^{2}\right)}^{1/2}$ along the diagonal and the angle θ = tan^−1^(*L*_*y*_/*L*_*x*_) between the diagonal and the domain side. The latter parameter determines the shape of the lattices, specifying the degree of distortion from ideal hexagonal geometry with θ = 60°.

The equilibrium period of the exchange‐frustrated SS was determined by the ratio of the second‐order (A) and fourth‐order (B) Heisenberg exchange terms, expressed as: $L_{H}=4\pi \sqrt{B/\left|A\right|}$. This parameter played a central role in the optimization scheme. The domain dimensions, *L*_*x*_ and *L*_*y*_, were directly related to *L*_H_ through a scaling factor. For instance, the equilibrium unit cells of the SL and S‐AL correspond to domains with mesh sizes of 71 × 128 × 1 and 56 × 128 × 1 cuboids, respectively. These meshes were chosen to maintain the ratio *N*_*y*_/*N*_*x*_ ≈ *L*_*y*_/*L*_*x*_, where *N*_*x*_ and *N*_*y*_ were the number of cuboids along the *x*‐ and *y*‐direction. This choice ensured that the cuboids were approximately square‐shaped and that the numbers of cuboids per unit length along the *x* and *y* axes were nearly identical. Accordingly, the optimal domain dimensions for SL were approximately *L*_*x*_ ≈ 1.41*L*_H_ and *L*_*y*_ ≈ 2.54*L*_H_, while for S‐AL they are *L*_*x*_ ≈ 1.3*L*_H_ and *L*_*y*_ ≈ 3*L*_H_. In the simulations, with *L*_H_ = 50 nm, the domain volumes representing the equilibrium SL and S‐AL unit cells were approximately 70.5 × 122.1 × 1 nm^3^ and 65 × 150 × 1 nm^3^, respectively.

Micromagnetic simulations were carried out with the following parameters: exchange stiffness along the *x*‐axis, $A_{x}$ = −10^−17^ Jm^−1^, and saturation magnetization, *M*_*s*_ = 400 kAm^−1^. The remaining interaction parameters in Equations (3) and (4) can be determined using the parameters α, β, *L*_H_, and *L*_D_. For details, the reader were referred to the Mumax Scripts.

### First‐Principles Calculations

Using ab initio spin‐polarized density functional theory (DFT) calculations within the Vienna Ab initio Simulation Package (VASP),^[^ ^69^, ^70^, ^71^ ^]^ the electronic and magnetic properties of the magnet/semiconductor heterostructure were investigated. In particular, this code was employed to determine the relaxed geometry of our slab construction. The asymmetric 2Fe/InSb(110) slab geometry, consisting of a bilayer Fe film on a nine‐layer thick InSb(110) substrate, was constructed using the experimental lattice constant of bulk InSb, 6.479 Å. A vacuum spacing of approximately 12 Å was maintained above and below the slab. Projector‐augmented wave (PAW) pseudopotentials^[^ ^72^, ^73^ ^]^ were used in conjunction with the Vosko‐Wilk‐Nusair (VWN) functional within the local spin density approximation (LSDA)^[^ ^74^ ^]^ for exchange‐correlation interactions. A 16 × 16 × 1 Γ‐centered k‐point mesh was considered for momentum‐space integration over the 2D Brillouin zone (2D‐BZ). A plane‐wave basis set with a cutoff energy of 500 eV was used for the expansion. The atomic positions within the slab were relaxed until the forces on all atoms in the magnetic Fe layers (surface and subsurface) and the first three substrate layers adjacent to the Fe/InSb interface converge to a value below 0.001 eVÅ^−1^.

### Calculation of Magnetic parameters

Following geometry relaxation, all magnetic parameters were extracted within the DFT code JuKKR,^[^ ^75^ ^]^ which utilized the full‐potential Korringa‐Kohn‐Rostoker (KKR) Green's function method.^[^ ^76^, ^77^ ^]^ This method offers an exact description of the atomic cell shape.^[^ ^78^, ^79^ ^]^ The slab consisted of 15 atomic layers (with 3 vacuum + 2 Fe layers + 7 InSb layers + 3 vacuum). This arrangement yields a vacuum spacing of approximately 7 Å on both the top and bottom surfaces of the slab. The momentum expansion of the Green's function had been truncated at *l*_max_ = 3. The same exchange‐correlation functional^[^ ^74^ ^]^ within the LSDA had been used. The self‐consistent calculations were performed using a 2D *k*‐points grid of 40 × 40 × 1, with a contour integration involving 38 complex energy points in the upper half‐plane, including five Matsubara poles. Self‐consistent spin‐polarized calculations, both with and without spin‐orbit coupling, were performed to converge the unit cell potential. With the converged potential, the pairwise Heisenberg exchange interactions and the DMI vectors were extracted using the infinitesimal rotation method^[^ ^80^, ^81^ ^]^ with a k‐mesh of a 200 × 200 × 1. Exchange and DMI interactions were truncated at a cutoff radius of approximately 10 Å, encompassing a total of 14 shells (seven intra‐layer and seven inter‐layer shells) for each Fe atom.

### Atomistic Spin‐Lattice Simulations

To elucidate the magnetic configuration of the system, the computed material parameters: the Heisenberg exchange interactions ($J_{ij}$), the DMI vector (**D**_*ij*_), and the single‐ion magnetocrystalline anisotropy (K) were employed, as elaborated in Note S3 (Supporting Information).^[^ ^31^ ^]^ These parameters serve as input for the extended Heisenberg model Hamiltonian, which takes the following form:

(7) $$\begin{array}{cc} H & =\;-\;\sum_{i>j}\;\left[\;J_{ij}{\hat{m}}_{i}\;\cdot \;{\hat{m}}_{j}+D_{ij}\;\cdot \;\left({\hat{m}}_{i}\;\times \;{\hat{m}}_{j}\right)\right]\;-\;K\sum_{i}{\left({\hat{m}}_{i}\;\cdot \;\hat{z}\right)}^{2}\; \end{array}$$

where *i* and *j* index the atomic sites within the domain, and $\hat{m}$ denotes a unit vector along the magnetic moment direction. As the model indicated, negative (positive) values of J correspond to antiferromagnetic (ferromagnetic) coupling, and their competition was crucial in determining the underlying ground state. It was important to note that the large magnetic unit cell, comprising eight Fe atoms and their long‐range interactions, necessitates a comprehensive exploration of the parameter space.

Now, the magnetic state of the system was determined by numerically minimizing Equation (7) using the Monte Carlo (MC) method as implemented in SPIRIT,^[^ ^67^ ^]^ a GPU‐accelerated atomistic code. To simulate the system with two magnetic layers, 2D domains composed of (80 × 80) × 2 magnetic atoms along *x* and *y* directions were constructed, with periodic boundary conditions imposed in most cases. To incorporate the effect of an external field, *B*_ext_, applied perpendicularly to the magnetic domain, a Zeeman term, $-\mu_{s}\sum_{i}B_{\mathrm{ext}}\cdot \hat{m_{i}}$, was included in the model Equation (7). To determine the zero‐temperature ground state in the absence of *B*_ext_, MC simulations were utilized by initializing the system with a random spin configuration at a high temperature of 100 K and then subsequently cooling it down to 0 K (10^−5^ in the code). These calculations were performed under open boundary conditions (OBC) to promote the formation of a cycloidal‐SS state. To further probe the equilibrium period, an SS state was imposed with varying periods within a finite domain, followed by cooling the system from high temperatures. The SS ground state served as the initial configuration for subsequent finite magnetic field simulations. This state was heated to a temperature of T = 50 K under an applied magnetic field and subsequently cooled down in 2 K steps. Importantly, regardless of the initial configuration, whether random or SS, the system invariably nucleates skyrmions and antiskyrmions at arbitrary positions within the domain under high temperature and magnetic field conditions. For each temperature step, 10^5^ MC steps were used for thermalization, followed by additional 10^5^ steps to calculate physical quantities such as magnetization, energy, and topological charge. Particular attention was paid to the lattice phases (SL and S‐AL), which were carefully relaxed in 0.5 K steps below 30 K. Additionally, the SL and S‐AL phases were subjected to multiple heating and cooling cycles within a 20 K temperature range under both OBC and PBC conditions, allowing to accurately determine their zero‐temperature energies.

## Conflict of Interest

The authors declare no conflict of interest.

## Supporting information

Supporting Information

## Data Availability

The data that support the findings of this article are not publicly available. The data are available from the authors upon reasonable request.
