# Supplementary material for: Paradoxical Topological Soliton Lattice in Anisotropic Frustrated Chiral Magnets
Source: Adv Sci (Weinh). 2025 Nov 6;13(5):e14568. doi: 10.1002/advs.202514568 (PMC12850401; doi:10.1002/advs.202514568)
Supplement: Supplementary file 1 — Supporting Information [file ADVS-13-e14568-s001.pdf]

# Supplemental material for: "Paradoxical Topological Soliton Lattice in Anisotropic Frustrated Chiral Magnets"

Sayan Banik,[1] Nikolai S. Kiselev,[2,*] and Ashis K. Nandy[1,†]

[1]School of Physical Sciences, National Institute of Science Education and Research, An OCC of Homi Bhabha National Institute, Jatni-752050, India

[2]Peter Grünberg Institute, Forschungszentrum Jülich, 52425 Jülich, Germany

## Supplementary Note 1| Taylor expansion of spin-lattice Hamiltonian

In this section, we present the derivations of the Hamiltonian describing a frustrated magnet in 2D.

**Heisenberg Exchange Interaction.** Given the symmetry properties of the lattice in the Heisenberg model, the coupling constants $\mathcal{J}_{ij}$ between the $i$-th and $j$-th atoms can be categorized into distinct shells defined by the lattice symmetry. Each site $i$ and its neighboring sites $k$ and $m$ have coupling constants $\mathcal{J}_{ik}$ and $\mathcal{J}_{im}$ that are equivalent under the symmetry operations of the point group corresponding to the crystal. This symmetry allows the position vectors $\mathbf{r}_{ik}$ and $\mathbf{r}_{im}$ to transform into each other.

We define a complete set of lattice sites that are symmetry-equivalent to form a shell, with each shell being labeled by an integer $s$. The coupling constant associated with each shell is denoted as $J_s$. For instance, the first shell (nearest neighbors) corresponds to $s = 1$, the second shell (next nearest neighbors) to $s = 2$, and so forth. Assuming that the vector $\mathbf{m}$ is a unit vector, the Heisenberg exchange interaction energy per one magnetic atom has the following form

$$\mathcal{H}_\mathrm{e} = -\sum_{i>j}^{N} \mathcal{J}_{ij}\mathbf{m}_i \cdot \mathbf{m}_j = \sum_{s}^{S} \frac{1}{4} J_s \sum_{k,l,m} \left[\mathbf{m}(\mathbf{r}) - \mathbf{m}(\mathbf{r} + a(k\mathbf{e}_x + l\mathbf{e}_y + m\mathbf{e}_y))\right]^2 \tag{1}$$

where $\mathbf{r}$ represents the position vector of the atom, and $k, l, m$ are sets of integer or half-integer indices that correspond to the shell $s$. We consider a crystal with a simple cubic Bravais lattice. Moving to the continuum limit, where the discrete spins $\mathbf{m}_i$ transit to a smoothly varying field $\mathbf{m}(\mathbf{r})$, we go beyond the conventional micromagnetic approximation and consider the higher-order terms in the series expansion. Let us consider a quasi-2D case, assuming that magnetization is homogeneous along the $z$-axis, $\mathbf{m} \equiv \mathbf{m}(x, y)$. In continuum approximation for cubic crystals, the Heisenberg exchange interaction with the terms up to fourth order can be written as[8]:

$$E_\mathrm{e} = \int_{\mathbb{R}^2} \left\{ \mathcal{A}\left[\left(\frac{\partial \mathbf{m}}{\partial x}\right)^2 + \left(\frac{\partial \mathbf{m}}{\partial y}\right)^2\right] + \mathcal{B}\left[\frac{\partial^2 \mathbf{m}}{\partial x^2} - \frac{\partial^2 \mathbf{m}}{\partial y^2}\right]^2 + \mathcal{C}\left[\frac{\partial^2 \mathbf{m}}{\partial x \partial y}\right]^2 \right\} t \, \mathrm{d}x \, \mathrm{d}y \tag{2}$$

where $t$ is the plate thickness. The derivation provides linear relations between micromagnetic parameters $\mathcal{A}$, $\mathcal{B}$, $\mathcal{C}$ and exchange constants $J_s$, connecting the spin-lattice model to continuum model with higher order term,

$$\mathcal{A} = \frac{1}{a}\sum_s \mathsf{a}_s J_s; \mathcal{B} = -a\sum_s \mathsf{b}_s J_s; \mathcal{C} = -a\sum_s \mathsf{c}_s J_s \tag{3}$$

where $a$ is the lattice constant. The positive coefficients $\mathsf{a}_s$, $\mathsf{b}_s$ and $\mathsf{c}_s$ depend on the crystal lattice type. We express $\mathcal{A}$ and $\mathcal{B}$, $\mathcal{C}$ in J/m and J·m units, respectively.

We now consider a simple cubic lattice with the lattice constant $a$ and coupling constants for the first four shells $\tilde{J}_{1,2,3,4}$ which reproduce the same material parameters $\mathcal{A}$, $\mathcal{B}$, $\mathcal{C}$. According to (3), these constants must satisfy the following system of equations (see Ref. 8 for details),

$$\begin{aligned}
\mathcal{A} &= \frac{1}{a}\Big(\frac{1}{2}\tilde{J}_1 + 2\tilde{J}_2 + 2\tilde{J}_3 + 2\tilde{J}_4\Big) \\
\mathcal{B} &= -a\Big(\frac{1}{96}\tilde{J}_1 + \frac{1}{24}\tilde{J}_2 + \frac{1}{24}\tilde{J}_3 + \frac{1}{6}\tilde{J}_4\Big) \\
\mathcal{C} &= -a\Big(\frac{1}{48}\tilde{J}_1 + \frac{1}{3}\tilde{J}_2 + \frac{7}{12}\tilde{J}_3 + \frac{1}{3}\tilde{J}_4\Big)
\end{aligned} \tag{4}$$

The model can be simplified by considering only the neighbors sitting along orthogonal directions. It means that we can exclude the interaction with other neighbors: $\tilde{J}_2 = 0$, $\tilde{J}_3 = 0$, and $\mathcal{C} = 2\mathcal{B}$. We refer to that model as *simplified effective model*. This model is depicted in Fig. **1**, see the Main text, where the next after-nearest neighbor exchange $J_1$ ($J_2$) is $\tilde{J}_1$ ($\tilde{J}_4$) in Eq. 4.

Let us show that in case of $\mathcal{C} = 2\mathcal{B}$, the integral (2) can be reduced to the exchange energy term Eq. (3) in the main text. For a smooth twice differentiable function $f$, one can prove the identity:

$$2\left(\frac{\partial^2 f}{\partial x \partial y}\right)^2 - 2\,\frac{\partial^2 f}{\partial x^2}\cdot\frac{\partial^2 f}{\partial y^2} = \frac{\partial^2}{\partial y^2}\left[\left(\frac{\partial f}{\partial x}\right)^2\right] + \frac{\partial^2}{\partial x^2}\left[\left(\frac{\partial f}{\partial y}\right)^2\right] - \frac{2\partial^2}{\partial x \partial y}\left(\frac{\partial f}{\partial x}\cdot\frac{\partial f}{\partial y}\right) \tag{5}$$

It is easy to show that the integration of every term on the right-hand side of (5) over $\mathbb{R}^2$ can be reduced to the boundary integral. For instance, the first term in the right-hand side of (5) can be written as

$$\int_{\mathbb{R}^2} \frac{\partial^2}{\partial y^2}\left[\left(\frac{\partial f}{\partial x}\right)^2\right] \mathrm{d}x\,\mathrm{d}y = \int_{\partial\mathbb{R}^2} \frac{\partial}{\partial y}\left[\left(\frac{\partial f}{\partial x}\right)^2\right] \mathrm{d}x,$$

where $\partial\mathbb{R}^2$ denotes the region's boundary. Without losing generality, in an extended system, when one can ignore the presence of edges and use periodic boundary conditions, such terms can always be set to zero. Thereby, for the smooth function $f$ defined in the whole $\mathbb{R}^2$ space, the integral of (5) can be set to zero

$$\int_{\mathbb{R}^2}\left\{2\left(\frac{\partial^2 f}{\partial x \partial y}\right)^2 - 2\,\frac{\partial^2 f}{\partial x^2}\cdot\frac{\partial^2 f}{\partial y^2}\right\}\mathrm{d}x\,\mathrm{d}y = 0 \tag{6}$$

Using the integral (6), one can show that for $\mathcal{C} = 2\mathcal{B}$ and assuming that every component of the magnetization vector field represents a continuous twice differentiable function, the term (2) can be written as

$$E = \int_{\mathbb{R}^2}\left\{\mathcal{A}\left[\left(\frac{\partial \mathbf{m}}{\partial x}\right)^2 + \left(\frac{\partial \mathbf{m}}{\partial y}\right)^2\right] + \mathcal{B}\left[\left(\frac{\partial^2 \mathbf{m}}{\partial x^2}\right)^2 + \left(\frac{\partial^2 \mathbf{m}}{\partial y^2}\right)^2\right]\right\} t\,\mathrm{d}x\,\mathrm{d}y \tag{7}$$

Finally, assuming that the exchange stiffness constants are not identical for orthogonal directions, the integral (7) can be written as the exchange energy term (3) in the main text.

Now, let's analyze the energy density for a spin spiral (SS) in the case of the frustrated magnet with $\mathcal{A} < 0$ and $\mathcal{B} > 0$. As follows from (4), the condition for $\mathcal{A} < 0$ is $\tilde{J}_4 < -\tilde{J}_1/4$. We will consider the solutions that, in the most general case, can be considered as conical spin spiral (cone-SS), Supplementary Fig. **S1**. In the absence of an external magnetic field and other potential energy terms as *e.g.* magnetocrystalline anisotropy, the solution can be written in the form of a flat-SS, $\mathbf{m}(\mathbf{r}) = (\cos(\mathbf{q}\cdot\mathbf{r}), \sin(\mathbf{q}\cdot\mathbf{r}), 0)$, where $\mathbf{r} = (x, y, z)$ is a position vector and $\mathbf{q}$ is the flat-SS wave vector. The energy density of flat-SS propagating along three different crystallographic directions, [111], [110], and [100], is given by,

$$\mathcal{E}_{\mathrm{s}} = \mathcal{A}_s q^2 + \begin{cases} \frac{2}{3}\mathcal{C}q^4 & (\mathbf{q} \parallel [111]), \\ (\mathcal{B} + \frac{1}{2}\mathcal{C})q^4 & (\mathbf{q} \parallel [110]), \\ 4\mathcal{B}q^4 & (\mathbf{q} \parallel [100]), \end{cases} \tag{8}$$

and the equilibrium wave vectors and the cone angle of a spiral in case of non-zero applied fields are as follows

$$\mathbf{q} = \frac{1}{2}\sqrt{\frac{-\mathcal{A}}{\mathcal{C}}}(\pm\hat{\mathbf{e}}_x \pm \hat{\mathbf{e}}_y \pm \hat{\mathbf{e}}_z), \quad \theta = \arccos(\frac{4M_s B_{\mathrm{ext}}\mathcal{C}}{3\mathcal{A}^2}), \tag{9}$$

$$\mathbf{q} = \frac{1}{\sqrt{2}}\sqrt{\frac{-\mathcal{A}}{2\mathcal{B}+\mathcal{C}}}(\pm\hat{\mathbf{e}}_x \pm \hat{\mathbf{e}}_y), \quad \theta = \arccos(\frac{M_s B_{\mathrm{ext}}(2\mathcal{B}+\mathcal{C})}{\mathcal{A}^2}), \tag{10}$$

$$\mathbf{q} = \frac{1}{2}\sqrt{\frac{-\mathcal{A}}{2\mathcal{B}}}(\pm\hat{\mathbf{e}}_\gamma), \quad \gamma \in \{x, y, z\}, \quad \theta = \arccos(\frac{8M_s B_{\mathrm{ext}}\mathcal{B}}{\mathcal{A}^2}). \tag{11}$$

For the simplified effective model ($\mathcal{C} = 2\mathcal{B}$), the energy densities of the SS states with equilibrium periods along three different directions:

$$\mathcal{E}_{\mathrm{eq}} = \begin{cases} -\dfrac{3\mathcal{A}^2}{16\mathcal{B}} & (\mathbf{q} \parallel [111]), \\ -\dfrac{\mathcal{A}^2}{8\mathcal{B}} & (\mathbf{q} \parallel [110]), \\ -\dfrac{\mathcal{A}^2}{16\mathcal{B}} & (\mathbf{q} \parallel [100]), \end{cases} \tag{12}$$

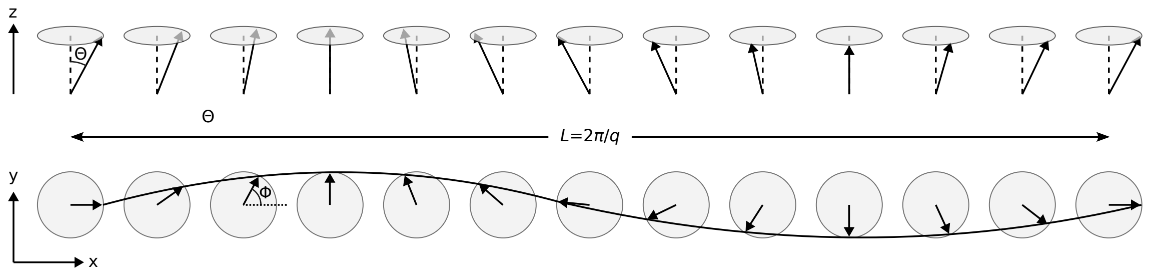


**Fig. S1 |** Conical SS with unit vector corresponding to each spin, $\mathbf{n}(\mathbf{r}) = [\cos(\mathbf{q}\cdot\mathbf{r})\sin(\Theta), \sin(\mathbf{q}\cdot\mathbf{r})\sin(\Theta), \cos(\Theta)]$. The polar angle $\Theta$, measured with respect to the $z$-axis, remains fixed at each lattice site. The planar view in the bottom panel depicts a spin spiral with a period of $L = 2\pi/q$, where the wavevector $\mathbf{q}$ is parallel to the $x$-axis. This cone-SS phase is subjected to an external magnetic field, $\mathbf{B}_{\text{ext}}$, applied along the $z$-axis. The in-plane component makes an angle $\Phi$ ($\equiv \mathbf{q}\cdot\mathbf{r}$) with the propagation direction.

Therefore, the energy comparison among the SSs with $\mathbf{q}$-vectors along three different directions is as follows:

$$\mathcal{E}_{[111]} < \mathcal{E}_{[110]} < \mathcal{E}_{[100]} \tag{13}$$

and thus in 2D, the lowest energy state is an SS with $\mathbf{q} \parallel [110]$.

Let's now consider the implementation of our simplified effective model in Mumax. We assume the following parameters are defined: saturation magnetization, $M_s$; exchange stiffness, $\mathcal{A}$; equilibrium period SS, $L_{\text{H}} = 2\pi/q$. The parameter $\mathcal{B}$ can be found from (10) using $L_{\text{H}}$:

$$\mathcal{B} = -\frac{\mathcal{A}L_{\text{H}}^2}{(4\pi)^2} \tag{14}$$

and the parameters $J_1 = \frac{\tilde{J}_1}{a}$ and $J_4 = \frac{\tilde{J}_4}{a}$ can be found from (4),

$$J_1 = \frac{8(a^2\mathcal{A} + 12\mathcal{B})}{3a^2} = \frac{8}{3}\mathcal{A} - \frac{2\mathcal{A}L_{\text{H}}^2}{\pi^2 a^2} = \frac{8}{3}\mathcal{A} - \frac{2}{\pi^2}\mathcal{A}\mathcal{N}^2 \tag{15}$$

$$\begin{aligned} J_4 = -\frac{(a^2\mathcal{A} + 48\mathcal{B})}{6a^2} &= -\frac{(a^2\mathcal{A} + 3\mathcal{A}L_{\text{H}}^2\pi^{-2})}{6a^2} \\ &= -\frac{1}{6}\mathcal{A} + \frac{1}{2\pi^2}\mathcal{A}\mathcal{N}^2 \end{aligned} \tag{16}$$

where $\mathcal{N} = L_{\text{H}}/a$ represents mesh density, i.e., the number of cuboids per one period of the SS. The parameter $\mathcal{N}$ is the internal parameter of a finite difference scheme.

From Eq. (14), the SS period can therefore be obtained as $L_{\text{H}} = 4\pi\sqrt{\frac{\mathcal{B}}{-\mathcal{A}}}$. For instance, with $\mathcal{A} = -10^{-17}$ J/m and $\mathcal{B} = 1.6 \times 10^{-34}$ J·m, the equilibrium period of SS is $L_{\text{H}} \sim 50$ nm, and the energetically preferred direction for its propagation is [110]. Finally, we introduce anisotropy parameter in exchange interactions $\alpha$ such that the Eqs. (4) reduces to,

$$\begin{aligned} \mathcal{A}_x &= \frac{1}{a}\Big(\frac{1}{2}\tilde{J}_{1x} + 2\tilde{J}_{4x}\Big), \\ \mathcal{A}_y &= \frac{\mathcal{A}_x}{\alpha} = \frac{1}{a}\Big(\frac{1}{2}\tilde{J}_{1y} + 2\tilde{J}_{4y}\Big), \\ \mathcal{B}_x &= -a\Big(\frac{1}{96}\tilde{J}_{1x} + \frac{1}{6}\tilde{J}_{4x}\Big), \\ \mathcal{B}_y &= \frac{\mathcal{B}_x}{\alpha} = -a\Big(\frac{1}{96}\tilde{J}_{1y} + \frac{1}{6}\tilde{J}_{4y}\Big) \end{aligned} \tag{17}$$

**Dzyaloshinskii-Moriya interaction.** Next, we consider the contribution of the Dzyaloshinskii-Moriya Interaction (DMI) in a system characterized by $C_{nv}$ symmetry. We define the magnitude of the DMI coupling constant $|\mathbf{D}_{ij}|$, representing the interaction strength between the $i$-th and $j$-th atoms. As observed in the symmetric properties of exchange interactions, the DMI coupling constants between the $i$-th site and its neighboring sites $k$ and $m$, denoted as $|\mathbf{D}_{ik}|$ and $|\mathbf{D}_{im}|$, exhibit equivalent magnitudes due to symmetry considerations.

The DMI in spin-lattice Hamiltonian can be written as follows:

$$\mathcal{H}_{\mathrm{D}} = -\sum_{i>j}^{N} |\mathbf{D}_{ij}| \mathbf{d}_{ij} \cdot [\mathbf{n}_i \times \mathbf{n}_j] = \sum_{s}^{S} D_s \sum_{k,l,m} \mathbf{d}_{k,l,m} [\mathbf{n}(\mathbf{r}) \times \mathbf{n}(\mathbf{r} + a(k\mathbf{e}_x + l\mathbf{e}_y + m\mathbf{e}_z))] \tag{18}$$

where $D_s$ represents the DMI coupling strength for $s$-th symmetry-defined shell. For consistency with the above, we consider only the first four shells.

Transitioning to the continuum limit ($\mathbf{n}_i \to \mathbf{n}(\mathbf{r})$), the Hamiltonian (18) with accuracy up to third-order terms can be written as follows:

$$E_{\mathrm{D}} = \int \mathcal{E}_{\mathrm{D}}\, d\mathbf{r} = \int \left( \mathcal{D}_1 \left( \Lambda_{xz}^{(x)} + \Lambda_{yz}^{(y)} \right) + \mathcal{D}_2 \left( \Lambda_{xz}^{(xxx)} + \Lambda_{yz}^{(yyy)} \right) + \mathcal{D}_3 \left( \Lambda_{xz}^{(xyy)} + \Lambda_{yz}^{(xxy)} \right) \right) d\mathbf{r} \tag{19}$$

The Lifshitz invariants, which describe the spatial modulation of the magnetization due to DMI, are defined as follows:

$$\begin{aligned} \Lambda_{ij}^{(k)} &= n_i \frac{\partial n_j}{\partial r_k} - n_j \frac{\partial n_i}{\partial r_k}, \\ \Lambda_{ij}^{(klm)} &= n_i \frac{\partial}{\partial r_k} \frac{\partial}{\partial r_l} \frac{\partial n_j}{\partial r_m} - n_j \frac{\partial}{\partial r_k} \frac{\partial}{\partial r_l} \frac{\partial n_i}{\partial r_m} \end{aligned} \tag{20}$$

The terms $\mathcal{D}_1$, $\mathcal{D}_2$, and $\mathcal{D}_3$ represent the constants of the first and third-order DMI terms, respectively, and can be related to the shell-based coupling constants of a simple cubic lattice through the relations:

$$\begin{aligned} \mathcal{D}_1 &= \frac{1}{a^2} \left( D_1 + 2\sqrt{2} D_2 + \frac{4}{\sqrt{3}} D_3 + 2 D_4 \right) \\ \mathcal{D}_2 &= \frac{1}{18} \left( 3D_1 + 6\sqrt{2} D_2 + 4\sqrt{3} D_3 + 24 D_4 \right) \\ \mathcal{D}_3 &= \frac{1}{6} \left( 3\sqrt{2} D_2 + 4\sqrt{3} D_3 \right) \end{aligned} \tag{21}$$

For consistency with the simplified effective model discussed above, we assume $D_2 = D_3 = 0$. Then, the above relations reduce to

$$\begin{aligned} \mathcal{D}_1 &= \frac{1}{a^2} \left( D_1 + 2D_4 \right), \\ \mathcal{D}_2 &= \frac{1}{6} D_1 + \frac{4}{3} D_4, \\ \mathcal{D}_3 &= 0, \end{aligned} \tag{22}$$

and thus the third term in (19) can be omitted. The constants $\mathcal{D}_1$ and $\mathcal{D}_2$ are expressed in units of [J/m$^2$] and [J], respectively. We omit the DMI term with $\mathcal{D}_2$ in the following. To justify the validity of such approximation, let us consider the case where the energy density functional includes only the leading Heisenberg exchange energy term and the third-order DMI term:

$$E = \int \left( \mathcal{A} \sum_{\alpha} \left( \frac{\partial \mathbf{n}}{\partial r_\alpha} \right)^2 + \mathcal{D}_2 \left( \Lambda_{xz}^{(xxx)} + \Lambda_{yz}^{(yyy)} \right) - M_{\mathrm{s}} \mathbf{B}_{\mathrm{ext}} \cdot \mathbf{n} \right) d\mathbf{r}\,. \tag{23}$$

Assuming the external magnetic field is applied along the $y$-axis, the following equation defines the SS:

$$\mathbf{n}(\mathbf{r}) = (\cos(\mathbf{q} \cdot \mathbf{r}) \sin(\Theta), \cos(\Theta), \sin(\mathbf{q} \cdot \mathbf{r}) \sin(\Theta)) \tag{24}$$

This SS corresponds to the scenario depicted in Fig. **S1**, with the axes exchanged as follows: $\{x, y, z\} \to \{x, -z, y\}$. According to (23), the energy density of such a configuration is

$$\mathcal{E} = (\mathcal{D}_2 q^3 + \mathcal{A} q^2) \sin^2(\Theta) - B_{\mathrm{ext}} \cos(\Theta), \tag{25}$$

It is seen that there is no equilibrium period of SS. It is important to emphasize that it is true irrespective of the sign of $\mathcal{A}$ and $\mathcal{D}_2$. The global minimum of (25) is $|q| \to \infty$ and the only metastable solution is $q = 0$. Since the actual values of $q$ are restricted between $-\pi/a$ and $+\pi/a$, where $a$ is the lattice constant, there is a critical value of $\mathcal{A} > \pi\mathcal{D}/a$ above which the global energy minimum of (23) is a ferromagnet (FM), *i.e.*, $q = 0$. When $\mathcal{A} < \pi\mathcal{D}/a$,

the global energy minimum corresponds to the latest possible value of $|q| = \pi/a$. Therefore, the only solutions to the model (23) are either FM or antiferromagnet. Consequently, the third-order DMI term, in competition with the leading Heisenberg energy term, cannot stabilize the SS as the ground state. The competition between high-order DMI and exchange may lead to a stable SS ground state only in the presence of a fourth-order Heisenberg exchange term. In this case, the energy density of a SS is $\mathcal{E} \sim \mathcal{B}q^4 + \mathcal{D}_2 q^3 + \mathcal{A}q^2$, allowing for a potential energy minimum at a finite, nonzero $q$. However, the contributions of higher-order terms, such as the fourth-order Heisenberg exchange and the third-order DMI, normally appear to be very weak compared to the other terms. In conclusion, we assume that higher-order DMI terms can be omitted in the first approximation.

Focusing on the first-order DMI term exclusively, we derive the following:

$$E_{\mathrm{D}} = \int \mathcal{E}_{\mathrm{D}}\, d\mathbf{r} = \int \left( \mathcal{D} \left( \Lambda_{xz}^{(x)} + \Lambda_{yz}^{(y)} \right) \right) d\mathbf{r}, \quad \text{where} \quad \mathcal{D} = \frac{D_1}{a^2} \tag{26}$$

In scenarios involving anisotropic systems, this expression simplifies to:

$$E_{\mathrm{D}} = \int \mathcal{E}_{\mathrm{D}}\, d\mathbf{r} = \int \left( \mathcal{D}_x \Lambda_{xz}^{(x)} + \mathcal{D}_y \Lambda_{yz}^{(y)} \right) d\mathbf{r} \tag{27}$$

**Isotropic model analysis.** Taking into account the transformation of the Heisenberg energy term presented in the Method section for the case $\mathcal{C} = 2\mathcal{B}$ the functional that describes isotropic magnetic system with frustrated exchange interaction and DMI is given by:

$$E(\mathbf{n}) = \int \left( \mathcal{A} \left[ \left( \frac{\partial \mathbf{m}}{\partial x} \right)^2 + \left( \frac{\partial \mathbf{m}}{\partial y} \right)^2 \right] + \mathcal{B} \left[ \left( \frac{\partial^2 \mathbf{m}}{\partial x^2} \right)^2 + \left( \frac{\partial^2 \mathbf{m}}{\partial y^2} \right)^2 \right] + \mathcal{D} \left[ \Lambda_{xz}^{(x)} + \Lambda_{yz}^{(y)} \right] - M_s \mathbf{B}_{\mathrm{ext}} \cdot \mathbf{n} \right) d\mathbf{r} \tag{28}$$

The energy density of a cycloidal-SS (24), with the wave vector $q$ aligned along the [100] crystallographic direction, as derived from equation (28) is

$$\mathcal{E} = (4\mathcal{B}q^4 + \mathcal{A}q^2 - \mathcal{D}q)\sin^2(\theta) - B_{\mathrm{ext}}\cos(\theta) \tag{29}$$

The only real solution for equation (29) is given by:

$$q = \frac{\sqrt[3]{6}\left(\sqrt{3}\sqrt{B^3(2A^3+27B)} + 9B^2\right)^{2/3} - 6^{2/3}AB}{12B\sqrt[3]{\left(\sqrt{3}\sqrt{B^3(2A^3+27B)} + 9B^2\right)}} \tag{30}$$

where $A = \dfrac{\mathcal{A}}{\mathcal{D}}$ and $B = \dfrac{\mathcal{B}}{\mathcal{D}}$. In the limiting case of $\mathcal{A} \to 0$, the solution simplifies to:

$$q = \frac{1}{2^{4/3}}\sqrt[3]{\frac{\mathcal{D}}{\mathcal{B}}}, \quad L_{\mathrm{D}} = 2\pi 2^{4/3}\sqrt[3]{\frac{\mathcal{B}}{\mathcal{D}}} \tag{31}$$

The energy density for a cycloidal-SS with $q$ aligned along [110] based on equation (28) is:

$$\mathcal{E} = (2\mathcal{B}q^4 + \mathcal{A}q^2 - \mathcal{D}q)\sin^2(\theta) - B_{\mathrm{ext}}\cos(\theta) \tag{32}$$

It is noted that the energy of the state described by equation (32) is lower than that of the SS in equation (29). The corresponding wave vector solution is:

$$q = \frac{\sqrt[3]{2}\left(\sqrt{3}\sqrt{B^3(4A^3+27B)} + 9B^2\right)^{2/3} - 2\sqrt[3]{3}AB}{2\cdot 6^{2/3}B\sqrt[3]{\left(\sqrt{3}\sqrt{B^3(4A^3+27B)} + 9B^2\right)}} \tag{33}$$

As $\mathcal{A} \to 0$, this solution reduces to:

$$q = \frac{1}{2\sqrt[3]{B}} = \frac{1}{2}\sqrt[3]{\frac{\mathcal{D}}{\mathcal{B}}}, \quad L_{\mathrm{D}} = 4\pi\sqrt[3]{\frac{\mathcal{B}}{\mathcal{D}}} \tag{34}$$

In the scenario where $\mathcal{D} \to 0$, the solution simplifies as referenced in equations (10) and (14):

$$q = \frac{1}{2}\sqrt{\frac{-\mathcal{A}}{\mathcal{B}}}, \quad L_{\mathrm{H}} = 4\pi\sqrt{\frac{\mathcal{B}}{-\mathcal{A}}} \tag{35}$$

Finally, introducing anisotropy into both the Heisenberg exchange and the DMI results in the following functional:

$$E(\mathbf{n}) = \int \left[ \left( \mathcal{A}_x \left(\frac{\partial \mathbf{m}}{\partial x}\right)^2 + \mathcal{A}_y \left(\frac{\partial \mathbf{m}}{\partial y}\right)^2 + \mathcal{B}_x \left(\frac{\partial^2 \mathbf{m}}{\partial x^2}\right)^2 + \mathcal{B}_y \left(\frac{\partial^2 \mathbf{m}}{\partial y^2}\right)^2 \right) + \left( \mathcal{D}_x \Lambda_{xz}^{(x)} + \mathcal{D}_y \Lambda_{yz}^{(y)} \right) - M_s \mathbf{B}_{\mathrm{ext}} \cdot \mathbf{n} \right] d\mathbf{r} \tag{36}$$

We define the anisotropies in the frustrated exchange as $\alpha$ and in the DMI as $\beta$, such that $\dfrac{\mathcal{A}_y}{\mathcal{A}_x} = \dfrac{\mathcal{B}_y}{\mathcal{B}_x} = \alpha$ and $\dfrac{\mathcal{D}_y}{\mathcal{D}_x} = \beta$, respectively. The energy density Eq. (36) is implemented within Mumax.

## Supplementary Note 2| Limiting cases within our Model

Our model, described by Eq. (36), demonstrates remarkable generality. It not only predicts the existence of the unprecedented skyrmion-antiskyrmion lattice (S-AL) phase as the energetically preferred ground state but also encompasses a broad spectrum of established theoretical and experimental scenarios, achieved by systematically exploring specific conditions in the model parameters.

### Case I: Isotropic frustrated magnet

We first investigate the role of exchange frustration by considering a simplified model where the DMI term in (36) is neglected ($\mathcal{D}_x = \mathcal{D}_y = 0$). Frustration in the exchange interactions is introduced by setting $\mathcal{A}_x = \mathcal{A}_y = \mathcal{A} < 0$ and $\mathcal{B}_x = \mathcal{B}_y = \mathcal{B} > 0$. In the absence of an external magnetic field, the model yields a cone-SS with an analytical period $L_{\mathrm{H}} \approx 50$ nm, as discussed in the main text. In this frustrated magnetic system, subjected to an external perpendicular field $h$, only two equilibrium phases are observed across the entire range of fields: the cone-SS phase, as depicted in Fig. **S1**, and the saturated FM. Figure **S2a** presents the energy density of the cone-SS as a function of its period at $h = 0.5$. The equilibrium period, determined to be approximately 50.1 nm, aligns well with the theoretical value. Notably, the period of the cone-SS remains independent of the external magnetic field.

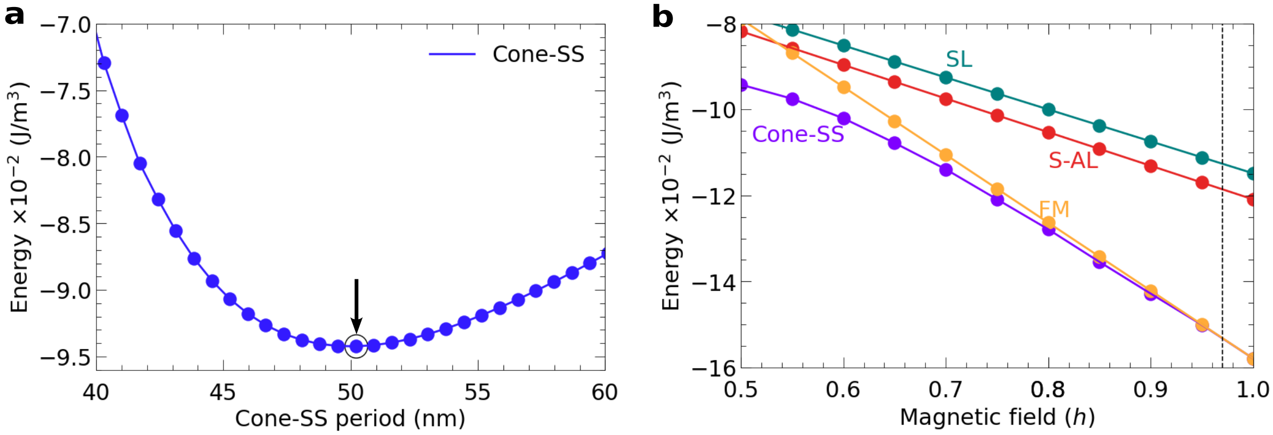


**Fig. S2 | Frustrated magnetic system with isotropic interactions: a**, Energy density plot as a function of the cone-SS period at perpendicular magnetic field $h = 0.5$. The arrow indicates the numerical equilibrium period corresponding to the lowest energy of cone-SS phase, $L_{\mathrm{H}} \approx 50.1$ nm. **b**, Energy density profiles as a function of $h$ for various magnetic phases: cone-SS, S-AL, SL, and FM. The vertical line indicates the second order phase boundary between cone-SS and FM phases. The optimized hexagonal S-AL and SL phases remain metastable throughout the entire magnetic field range.

In Fig. **S2b**, we present the energy density profiles for the cone-SS, FM, S-AL, and skyrmion lattice (SL) phases as a function of the external magnetic field $h$. Two distinct regions, corresponding to the cone-SS and FM phases, are

separated by a second-order phase boundary and identified as the energetically favored ground states across the entire magnetic field range. In isotropic frustrated magnets, these two phases are commonly observed as ground states under specific magnetic field regions, as earlier demonstrated by the phase diagram in Ref.[5]. Our model, in the absence of magnetocrystalline anisotropy, accurately reproduces this limiting behavior. Although the SL and S-AL possess equilibrium periods, they always occupy higher energy states than the cone-SS and saturated FM phases, irrespective of the applied magnetic field $h$. Notably, even in the absence of DMI, the optimized S-AL exhibits lower energy than the optimized SL in the presence of exchange frustration.

**Case II: Conventional chiral magnet**

The second limiting case considers conventional chiral magnets with isotropic DMI and FM exchange interactions, as described by our model (36) with $\mathcal{A} = \mathcal{A}_x = \mathcal{A}_y > 0$ and $\mathcal{B} = 0$. The DMI here lifts the degeneracy between clockwise and anti-clockwise SSs, resulting in the formation of a chiral phase. The DMI term in model (36) stabilizes a cycloidal-SS phase, consistent with the behavior observed in two-dimensional chiral magnets with interfacial DMI. Without an external magnetic field, the ground state solution here is a right-handed cycloidal-SS, whose equilibrium period is exclusively governed by $4\pi\frac{\mathcal{A}}{|\mathcal{D}|}$[7]. As a specific example, for $\mathcal{A} = 10^{-17}$ J/m and $\mathcal{D} = 12.5 \times 10^{-19}$ J/m$^2$, the analytical period of the cycloidal-SS solution is approximately 100 nm.

A common scenario involves the transition of the cycloidal-SS state to a triangular lattice of magnetic solitons under an external magnetic field $h$ applied perpendicular to the 2D plate. However, these lattices are composed of a single type of soliton: skyrmions for $\mathcal{D}_x = \mathcal{D}_y$ or antiskyrmions for $\mathcal{D}_x = -\mathcal{D}_y$[3]. Considering $\mathcal{D}_x = \mathcal{D}_y$, Fig. **S3a** presents the energy density landscape as a function of $h$, revealing a stable SL phase bounded by two first-order phase transition lines at critical fields $h \sim 0.11$ and $h \sim 0.39$. The lattice is a regular hexagonal lattice of axisymmetric skyrmions. Two critical field values define the phase boundaries separating the cycloidal-SS, SL, and saturated FM phases. It is noteworthy that both the S-AL and antiskyrmion lattice phases are unstable in this DMI arrangement.

Additionally, we consider the case of anisotropic DMI, characterized by $\mathcal{D}_x \neq \mathcal{D}_y$ and controlled by the parameter $\beta$. In this anisotropic scenario, we identify the equilibrium SS period and hexagonal SL solution by systematically adjusting the domain size in our simulations. The energy density profiles in Fig. **S3b** reveal the presence of minima, which correspond to the system's minimum energy configuration for different $\beta$ values. These simulations, conducted at zero external field ($h = 0$), demonstrate the cycloidal-SS state as the ground state configuration. Our analysis reveals a critical dependence of the SL phase on DMI anisotropy. Below a critical value of $\beta \approx 0.8$, the SL phase becomes energetically unfavorable and disappears. This critical behavior is evident in the domain size dependence: for domains exceeding the critical sizes (marked by red stars), the SL phase destabilizes and transitions into a stable SS solution. This instability is analogous to the behavior observed in monoaxial chiral magnets, characterized by the absence of DMI along one direction, as described in Ref.[4]. In such systems, the energetically favored magnetic states are typically cycloidal-SS and saturated FM. The critical behavior of $\beta$ in destabilizing the SL remains evident, even in the presence of a finite external field ($h = 0.35$), as shown in Fig. **S3c**.

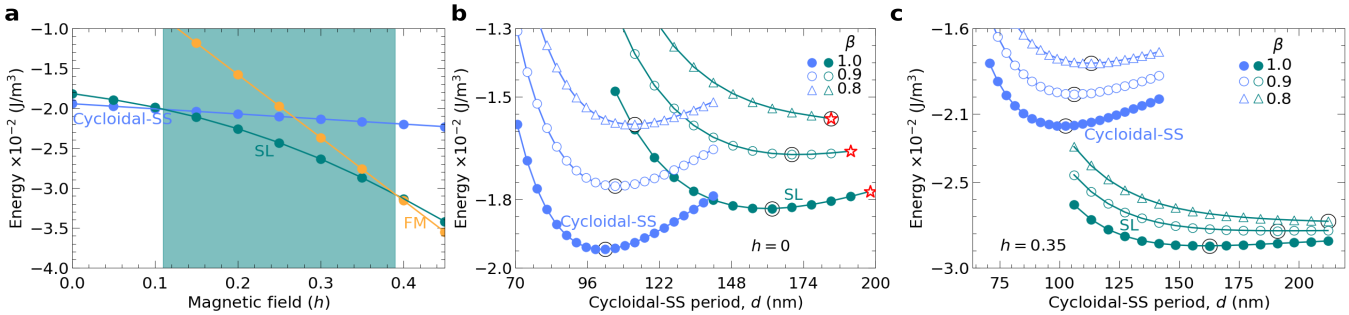


**Fig. S3 | Magnetic phases in conventional chiral magnets.** **a**, Energy density lines of cycloidal-SS, hexagonal SL, and saturated FM as a function of magnetic field $h$. The colored region, bounded by two first-order phase transition lines, represents the equilibrium SL phase. This phase exists between the cycloidal-SS and saturated FM phases. **b**, at $h = 0$, and **c**, at $h = 0.35$, depict energy density variations with respect to the SS period and *core-to-core* distances $d$ between two skyrmions for different DMI anisotropy parameter, $\beta$. The plot identifies regions of minimal energy (black circles) and points of instability for the SL (red asterisks).

**Case III: Isotropic frustrated chiral magnet.**

There exist isotropic systems, e.g., ultrathin chiral magnets, which can exhibit both frustrated exchange and DMI interactions. This represents the third limiting case within our model. The corresponding phase diagram, obtained within our model by setting $\alpha = \beta = 1$, is already presented in Fig. **3a** of the main text. However, the stability

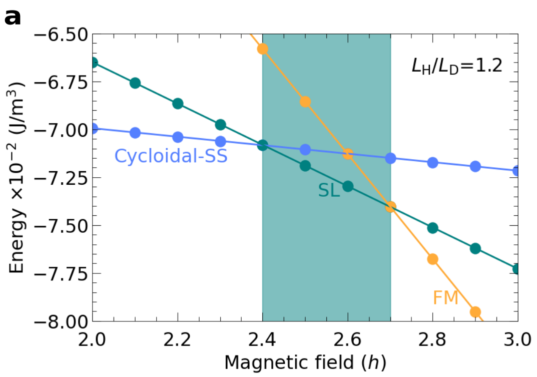


**Fig. S4 | Energy density profile for isotropic frustrated chiral magnet. a,** Energetically favored three phases: cycloidal-SS, hexagonal SL, and saturated FM. The SL phase is bounded by two first-order phase transition critical fields: $h = 2.4$ (SS to SL) and $h = 2.7$ (SL to FM).

of the SL phase as the ground state is critically dependent upon a delicate balance between exchange and DMI energies. This energy balance can be controlled by the ratio $L_{\mathrm{H}}/L_{\mathrm{D}}$. When the DMI strength becomes sufficiently strong compared to the exchange energy, we observe a first-order phase transition. In this transition, the cycloidal-SS transforms into the hexagonal SL phase. In this limiting case, exchange frustration often stabilizes the SS state in such systems, but without a preferred rotational sense. The DMI lifts this degeneracy, selecting a specific rotational direction–the cycloidal-SS phase. To model this isotropic case, we set the exchange interactions to $\mathcal{A} = \mathcal{A}_x = \mathcal{A}_y < 0$ and $\mathcal{B} = \mathcal{B}_x = \mathcal{B}_y > 0$, and the DMI strength to $|\mathcal{D}| = |\mathcal{D}_x| = |\mathcal{D}_y|$. By tuning the DMI strength to a high value ($L_{\mathrm{H}}/L_{\mathrm{D}} = 1.2$), we observe a typical phase sequence of cycloidal-SS, hexagonal SL, and saturated FM states as the magnetic field $h$ is increased, as shown in Fig. **S4**. Within the field range from $h = 2.4$ to $h = 2.7$, the system exhibits the SL phase. As demonstrated in previous studies[1,2,6], atomistic spin-lattice simulations can also accurately capture the general behavior of such isotropic chiral magnets and other magnetic phases, including the SL phase.

## Supplementary Note 3| 2Fe/InSb(110) a detailed analysis

### Magnetic characterization of the 2D film within ab initio electronic structure calculations.

The relaxed film geometry, obtained through structural optimization, serves as the basis for subsequent *ab initio* calculations. The magnetic moments of each Fe atom are tabulated in Table **S1**. The initial four values pertain to the top Fe layer atoms, whereas the subsequent four are associated with the bottom Fe layer, as indicated by the numbering scheme in Figs. **S5a** and **b**. To ensure consistency, we present values obtained from both VASP and KKR calculations. While generally consistent, a slight difference is noticeable, with KKR values typically exceeding those calculated using VASP. As described in the main text, the atomistic spin-lattice simulations employed an average magnetic moment of 2.71 $\mu_{\mathrm{B}}$ per Fe atom and an out-of-plane magnetocrystalline anisotropy of 0.6 meV per Fe atom, values obtained from KKR calculations.

| Fe atom | Magnetic mom. in $\mu_B$ KKR (VASP) | Average ($\mu_B$) KKR (VASP) | $\mathcal{K}$ (meV/Fe atom) |
|---|---|---|---|
| Fe 1 | 2.76 (2.69) | 2.71 (2.61) | 0.6 |
| Fe 2 | 2.80 (2.76) | | |
| Fe 3 | 3.07 (2.92) | | |
| Fe 4 | 3.06 (2.90) | | |
| Fe 5 | 2.46 (2.42) | | |
| Fe 6 | 2.41 (2.33) | | |
| Fe 7 | 2.65 (2.49) | | |
| Fe 8 | 2.50 (2.39) | | |

**Table S1 | Magnetic moments of Fe atoms and uniaxial magnetocrystalline anisotropy.** Each Fe atom has different magnetic moments, which is a consequence of the anisotropic interactions present in our system. The magnetic moments are expressed in the units of $\mu_B$ while $\mathcal{K}$ is the out-of-plane uniaxial magnetocrystalline anisotropy.

The magnetic heterostructure, featuring two distinct magnetic layers (Fig. **S5** and also in Figs. **4a** and **b** in the main text), exhibits a rich complexity arising from eight unique Fe atomic configurations with diverse local environments. This structural uniqueness, arising from low symmetry, results in significant variations in exchange and DMI parameters. The corresponding interaction parameters for each configuration, determined through KKR calculations, are tabulated in Tables **S2-S9**. It is crucial to note that the intricate nature of these interfacial systems results in a remarkably large parameter space. Furthermore, long-range interactions between magnetic atoms significantly influence their frustrated behavior. For further clarity, Figs. **S5c** and **d** (upper panels) provide a detailed visualization

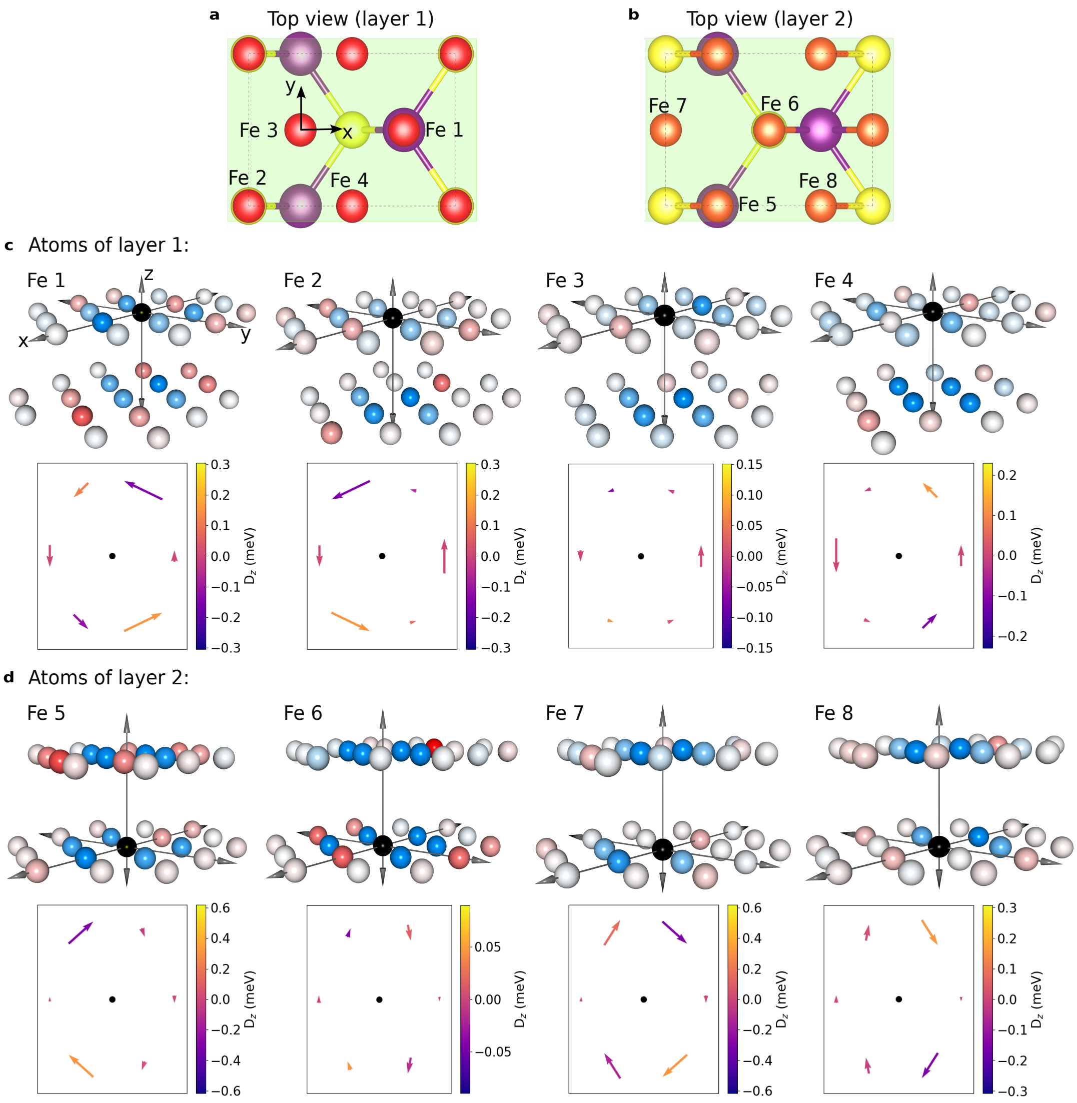


**Fig. S5 | Inequivalent Fe atoms in the heterostructure and corresponding magnetic interactions.** Top views of the two magnetic layers, each consisting of four Fe atoms: layer 1 in **a** and layer 2 in **b**. The distinct local environments of the eight Fe atoms, arising from the interfacial structure, result in anisotropic Fe−Fe interactions. For instance, considering the adjacent InSb(110) semiconductor layer, Fe3 exhibits a pronounced asymmetry in its nearest-neighbor coordination, with a clear absence of atoms in the $-x$ direction compared to the $+x$ direction. Furthermore, In atoms are present in the $\pm y$ directions, while the $\pm x$ directions lack In atoms. Similar asymmetric coordination patterns are observed for other Fe atoms. This broken symmetry at their coordination manifests in the material interaction parameters (exchange and DMI), leading to a pronounced anisotropic behavior, as detailed in panels **c,** and **d**.

of the exchange coupling strengths between Fe atoms within the first and second layers, respectively. Each sphere is color-coded according to the magnitude of exchange coupling strengths ($\mathcal{J}$), with more saturated hues denoting stronger interactions. Reference Fe atoms are highlighted in black, with neighboring atoms color-coded to indicate

the nature of the exchange interaction: blue for FM and red for antiferromagnetic. This visualization comprehensively maps both intra- and inter-layer couplings across the entire system. For a detailed visualization of the DMI orientation for the first two neighbors of each Fe atom, we present vector plots in the lower panels of Figs. **S5c** and **d**. The color bar at right in these plots represents the strength of the DMI's $z$-component, which is smaller than the other components. This is non-negligible due to the surface roughness and the anisotropic substrate environment. Importantly, these visualizations emphasize the presence of anisotropy in both exchange and DMI.

| Interactions for atom: Fe 1 | | | | | | | |
|---|---|---|---|---|---|---|---|
| | Neighbor atom (Fe) | $J_{ij}$ (meV) | $D_x$ (meV) | $D_y$ (meV) | $D_z$ (meV) | $\|\mathbf{D}\|$ (meV) | $\|\mathbf{R}\|$ (a) |
| Intra-layer interactions | 2 | 6.097 | 1.230 | 0.397 | 0.305 | 1.327 | 0.433 |
| | 2 | 6.097 | -1.230 | 0.397 | -0.305 | 1.327 | 0.433 |
| | 4 | 9.975 | 0.471 | -0.313 | -0.229 | 0.610 | 0.439 |
| | 4 | 9.975 | -0.471 | -0.313 | 0.229 | 0.610 | 0.439 |
| | 3 | 18.235 | 0.000 | 0.249 | -0.000 | 0.249 | 0.507 |
| | 3 | -4.662 | 0.000 | -0.473 | 0.000 | 0.473 | 0.507 |
| | 1 | -3.532 | -0.269 | -0.000 | -0.102 | 0.288 | 0.707 |
| | 1 | -3.532 | 0.269 | 0.000 | 0.102 | 0.288 | 0.707 |
| | 2 | 1.579 | -0.009 | 0.055 | -0.081 | 0.098 | 0.829 |
| | 2 | 1.579 | 0.009 | 0.055 | 0.081 | 0.098 | 0.829 |
| | 4 | 1.168 | 0.070 | 0.215 | -0.058 | 0.233 | 0.832 |
| | 4 | 1.168 | -0.070 | 0.215 | 0.058 | 0.233 | 0.832 |
| | 3 | -1.915 | -0.132 | -0.055 | 0.097 | 0.172 | 0.870 |
| | 3 | -1.915 | 0.132 | -0.055 | -0.097 | 0.172 | 0.870 |
| | 3 | 0.512 | -0.140 | -0.126 | 0.214 | 0.286 | 0.870 |
| | 3 | 0.512 | 0.140 | -0.126 | -0.214 | 0.286 | 0.870 |
| | 1 | 0.074 | 0.000 | -0.034 | -0.000 | 0.034 | 1.000 |
| | 1 | 0.074 | -0.000 | 0.034 | 0.000 | 0.034 | 1.000 |
| Inter-layer interactions | 6 | 9.022 | -0.000 | -0.323 | -0.000 | 0.323 | 0.284 |
| | 7 | 29.892 | -0.000 | 0.628 | 0.000 | 0.628 | 0.353 |
| | 8 | 8.547 | 0.067 | 0.349 | 0.345 | 0.496 | 0.433 |
| | 8 | 8.547 | -0.067 | 0.349 | -0.345 | 0.496 | 0.433 |
| | 5 | -4.370 | -0.124 | 0.119 | 0.163 | 0.237 | 0.639 |
| | 5 | -4.370 | 0.124 | 0.119 | -0.163 | 0.237 | 0.639 |
| | 5 | -6.111 | 0.186 | -0.071 | 0.042 | 0.204 | 0.639 |
| | 5 | -6.111 | -0.186 | -0.071 | -0.042 | 0.204 | 0.639 |
| | 6 | 0.202 | 0.029 | 0.047 | 0.098 | 0.113 | 0.762 |
| | 6 | -9.165 | -0.000 | -0.016 | 0.000 | 0.016 | 0.762 |
| | 6 | 0.202 | -0.029 | 0.047 | -0.098 | 0.113 | 0.762 |
| | 7 | -4.700 | -0.000 | -0.033 | -0.000 | 0.033 | 0.790 |
| | 7 | 0.378 | -0.204 | 0.185 | -0.346 | 0.442 | 0.790 |
| | 7 | 0.378 | 0.204 | 0.185 | 0.346 | 0.442 | 0.790 |
| | 6 | -0.878 | 0.013 | 0.100 | 0.127 | 0.163 | 1.040 |
| | 6 | -0.878 | -0.013 | 0.100 | -0.127 | 0.163 | 1.040 |
| | 8 | 0.438 | 0.124 | 0.076 | -0.163 | 0.218 | 1.090 |
| | 8 | 0.438 | -0.124 | 0.076 | 0.163 | 0.218 | 1.090 |

**Table S2 | Heisenberg exchange interaction and DMI parameters for Fe 1 atom.** Both intra- and inter-layer interactions are taken into account. The distance between the neighbors is given in the units of the experimental lattice constant of InSb, 6.479 Å.

| Interactions for atom: Fe 2 | | | | | | | |
|---|---|---|---|---|---|---|---|
| | Neighbor atom (Fe) | $J_{ij}$ (meV) | $D_x$ (meV) | $D_y$ (meV) | $D_z$ (meV) | $\|\mathbf{D}\|$ (meV) | $\|\mathbf{R}\|$ (a) |
| Intra-layer interactions | 1 | 6.097 | -1.230 | -0.397 | -0.305 | 1.327 | 0.433 |
| | 1 | 6.097 | 1.230 | -0.397 | 0.305 | 1.327 | 0.433 |
| | 3 | 7.625 | 0.208 | 0.040 | 0.150 | 0.259 | 0.444 |
| | 3 | 7.625 | -0.208 | 0.040 | -0.150 | 0.259 | 0.444 |
| | 4 | -0.545 | -0.000 | -0.461 | -0.000 | 0.461 | 0.508 |
| | 4 | -4.880 | 0.000 | 0.746 | -0.000 | 0.746 | 0.508 |
| | 2 | -5.217 | -0.245 | 0.000 | -0.324 | 0.406 | 0.707 |
| | 2 | -5.217 | 0.245 | -0.000 | 0.324 | 0.406 | 0.707 |
| | 1 | 1.579 | -0.009 | -0.055 | -0.081 | 0.098 | 0.829 |
| | 1 | 1.579 | 0.009 | -0.055 | 0.081 | 0.098 | 0.829 |
| | 3 | -0.341 | -0.106 | -0.151 | 0.018 | 0.185 | 0.835 |
| | 3 | -0.341 | 0.106 | -0.151 | -0.018 | 0.185 | 0.835 |
| | 4 | -2.272 | -0.039 | 0.074 | -0.058 | 0.102 | 0.871 |
| | 4 | 0.140 | 0.070 | 0.117 | -0.081 | 0.158 | 0.871 |
| | 4 | 0.140 | -0.070 | 0.117 | 0.081 | 0.158 | 0.871 |
| | 4 | -2.272 | 0.039 | 0.074 | 0.058 | 0.102 | 0.871 |
| | 2 | -1.321 | -0.000 | 0.356 | 0.000 | 0.356 | 1.000 |
| | 2 | -1.321 | 0.000 | -0.356 | -0.000 | 0.356 | 1.000 |
| Inter-layer interactions | 5 | 16.590 | 0.000 | 0.131 | -0.000 | 0.131 | 0.300 |
| | 8 | 39.792 | -0.000 | 0.408 | 0.000 | 0.408 | 0.342 |
| | 7 | 10.437 | -0.248 | 0.037 | -0.037 | 0.253 | 0.424 |
| | 7 | 10.437 | 0.248 | 0.037 | 0.037 | 0.253 | 0.424 |
| | 6 | 0.354 | 0.111 | -0.298 | -0.056 | 0.323 | 0.624 |
| | 6 | 0.354 | -0.111 | -0.298 | 0.056 | 0.323 | 0.624 |
| | 6 | -0.438 | 0.044 | -0.027 | -0.083 | 0.098 | 0.624 |
| | 6 | -0.438 | -0.044 | -0.027 | 0.083 | 0.098 | 0.624 |
| | 5 | 0.688 | -0.040 | -0.015 | 0.030 | 0.052 | 0.768 |
| | 5 | 0.688 | 0.040 | -0.015 | -0.030 | 0.052 | 0.768 |
| | 5 | -9.858 | 0.000 | -0.265 | -0.000 | 0.265 | 0.768 |
| | 8 | -6.573 | 0.000 | 0.109 | 0.000 | 0.109 | 0.785 |
| | 8 | -0.703 | -0.185 | -0.104 | -0.012 | 0.213 | 0.785 |
| | 8 | -0.703 | 0.185 | -0.104 | 0.012 | 0.213 | 0.785 |
| | 5 | -0.484 | 0.013 | 0.097 | 0.039 | 0.105 | 1.044 |
| | 5 | -0.484 | -0.013 | 0.097 | -0.039 | 0.105 | 1.044 |
| | 7 | 0.486 | 0.019 | 0.086 | -0.113 | 0.144 | 1.086 |
| | 7 | 0.486 | -0.019 | 0.086 | 0.113 | 0.144 | 1.086 |

**Table S3 | Heisenberg exchange interaction and DMI parameters for Fe 2 atom.** Both intra- and inter-layer interactions are taken into account. The distance between the neighbors is given in the units of the experimental lattice constant of InSb, 6.479 Å.

**Magnetic characterization of the heterostructure through atomistic spin-lattice simulations**

The Hamiltonian describing the magnetic ground state of the system is provided in Eq. (6), as described in the Methods section. To isolate the role of exchange frustration, we have initially removed all other interaction parameters, including DMI, magnetocrystalline anisotropy, and the Zeeman energy terms from the full Hamiltonian within our Monte Carlo (MC) simulations. This approach allowed us to investigate whether competing exchange interactions alone can drive the system to an SS state solution. Remarkably, this simulation resulted in the spontaneous formation of an SS state driven entirely by competing exchange interactions *i.e.*, the exchange frustration. Analysis of the simulation data reveals an exchange frustration-driven spiral with a period of approximately 2.6 nm, as shown in Fig. **S6a**. Heisenberg exchange-frustrated systems with an SS order invariably acquire a chiral behavior (a distinct rotational sense) when DMI interactions are introduced into the Hamiltonian. Crucially, the DMI interaction dictates the handedness of the chiral order and exerts a subtle influence on the spiral's period, as shown in Fig. **S6b**. In this cycloidal-SS state, the introduction of out-of-plane magnetocrystalline anisotropy further can reduce the SS period. As shown in Fig. **S6b**, the SS period exhibits a notable decrease of approximately 0.3 nm compared to the case driven solely by exchange frustration.

**Spontaneous nucleation of both elongated skyrmions and antiskyrmions.**

Building upon the findings (cluster of skyrmions and antiskyrmions) presented in Extended Data Fig. **5**, we here investigate the stability of metastable states characterized by the coexistence of skyrmions and antiskyrmions. To

| Interactions for atom: Fe 3 | | | | | | | |
|---|---|---|---|---|---|---|---|
| | Neighbor atom (Fe) | $J_{ij}$ (meV) | $D_x$ (meV) | $D_y$ (meV) | $D_z$ (meV) | $\lvert\mathbf{D}\rvert$ (meV) | $\lvert\mathbf{R}\rvert$ (a) |
| Intra-layer interactions | 2 | 7.625 | 0.208 | -0.040 | 0.150 | 0.259 | 0.444 |
| | 2 | 7.625 | -0.208 | -0.040 | -0.150 | 0.259 | 0.444 |
| | 1 | 18.235 | -0.000 | -0.249 | 0.000 | 0.249 | 0.507 |
| | 1 | -4.662 | -0.000 | 0.473 | -0.000 | 0.473 | 0.507 |
| | 4 | 4.395 | 0.207 | 0.035 | -0.259 | 0.334 | 0.612 |
| | 4 | 2.537 | -0.214 | 0.057 | -0.028 | 0.223 | 0.612 |
| | 4 | 2.537 | 0.214 | 0.057 | 0.028 | 0.223 | 0.612 |
| | 4 | 4.395 | -0.207 | 0.035 | 0.259 | 0.334 | 0.612 |
| | 3 | -0.103 | -0.357 | 0.000 | 0.356 | 0.505 | 0.707 |
| | 3 | -0.103 | 0.357 | -0.000 | -0.356 | 0.505 | 0.707 |
| | 2 | -0.341 | 0.106 | 0.151 | -0.018 | 0.185 | 0.835 |
| | 2 | -0.341 | -0.106 | 0.151 | 0.018 | 0.185 | 0.835 |
| | 1 | 0.512 | 0.140 | 0.126 | -0.214 | 0.286 | 0.870 |
| | 1 | 0.512 | -0.140 | 0.126 | 0.214 | 0.286 | 0.870 |
| | 1 | -1.915 | 0.132 | 0.055 | -0.097 | 0.172 | 0.870 |
| | 1 | -1.915 | -0.132 | 0.055 | 0.097 | 0.172 | 0.870 |
| | 3 | -0.795 | -0.000 | -0.202 | -0.000 | 0.202 | 1.000 |
| | 3 | -0.795 | 0.000 | 0.202 | 0.000 | 0.202 | 1.000 |
| Inter-layer interactions | 6 | 17.500 | -0.000 | -0.438 | 0.000 | 0.438 | 0.333 |
| | 7 | 44.387 | 0.000 | 0.384 | -0.000 | 0.384 | 0.334 |
| | 5 | 12.908 | 0.166 | -0.016 | -0.044 | 0.172 | 0.508 |
| | 5 | 12.908 | -0.166 | -0.016 | 0.044 | 0.172 | 0.508 |
| | 8 | 2.947 | 0.023 | 0.158 | -0.053 | 0.168 | 0.697 |
| | 8 | -2.512 | -0.033 | -0.118 | 0.148 | 0.192 | 0.697 |
| | 8 | 2.947 | -0.023 | 0.158 | 0.053 | 0.168 | 0.697 |
| | 8 | -2.512 | 0.033 | -0.118 | -0.148 | 0.192 | 0.697 |
| | 6 | 2.375 | -0.000 | -0.006 | -0.000 | 0.006 | 0.782 |
| | 6 | 0.874 | -0.043 | 0.010 | 0.060 | 0.074 | 0.782 |
| | 6 | 0.874 | 0.043 | 0.010 | -0.060 | 0.074 | 0.782 |
| | 7 | 0.930 | 0.203 | 0.002 | -0.015 | 0.203 | 0.782 |
| | 7 | 0.930 | -0.203 | 0.002 | 0.015 | 0.203 | 0.782 |
| | 5 | -1.394 | -0.030 | -0.021 | 0.102 | 0.109 | 0.871 |
| | 5 | -1.394 | 0.030 | -0.021 | -0.102 | 0.109 | 0.871 |
| | 7 | 2.023 | 0.000 | -0.009 | -0.000 | 0.009 | 1.054 |

**Table S4 | Heisenberg exchange interaction and DMI parameters for Fe 3 atom.** Both intra- and inter-layer interactions are taken into account. The distance between the neighbors is given in the units of the experimental lattice constant of InSb, 6.479 Å.

nucleate topological magnetic spin textures, our MC simulations begin with a random magnetic configuration and subsequently undergo a systematic process of simulated annealing at a finite magnetite field[9]. In this process, the simulation domain involves two magnetic layers, each consisting of an $80 \times 80 \times 1$ array of spins, subjected to a perpendicular magnetic field of approximately 0.5 Tesla. The total number of spins within the domain is 12800. As presented in Fig. **S7**, We observe the nucleation and subsequent evolution of topological charges with a magnitude of unity. This leads to a domain exclusively composed of elongated skyrmions and antiskyrmions. Notably, at zero temperatures, as depicted in Fig. **S7d**, skyrmions and antiskyrmions are distinctly identified by red and blue boxes, respectively. It is crucial to note that the true ground state at this magnetic field is the cone-SS phase, as evident in the magnetic phase diagram shown in Fig. **4c** of the main text. Within this cone-SS background, a metastable cluster of skyrmions and antiskyrmions coexists. While the precise number of skyrmions and antiskyrmions may vary, our simulations have not revealed any other topological spin textures with a topological charge greater than unity. To further investigate this coexisting behavior, we employ a systematic simulated annealing protocol within MC simulations under a finite magnetic field. Now, for these simulations, we initialize the system with a cycloidal-SS configuration. Figure **S8** illustrates the results of our simulations, demonstrating a metastable state characterized by the coexistence of topologically distinct spin textures–skyrmions and antiskyrmions–within a background of cone-SS. These findings provide critical support for the congruence between our micromagnetic and atomistic models, further validating our predictions for this novel magnetic phase in a real-material system.

| Interactions for atom: Fe 4 | | | | | | | |
|---|---|---|---|---|---|---|---|
| | Neighbor atom (Fe) | $J_{ij}$ (meV) | $D_x$ (meV) | $D_y$ (meV) | $D_z$ (meV) | $\|\mathbf{D}\|$ (meV) | $\|\mathbf{R}\|$ (a) |
| Intra-layer interactions | 1 | 9.975 | -0.471 | 0.313 | 0.229 | 0.610 | 0.439 |
| | 1 | 9.975 | 0.471 | 0.313 | -0.229 | 0.610 | 0.439 |
| | 2 | -0.545 | 0.000 | 0.461 | 0.000 | 0.461 | 0.508 |
| | 2 | -4.880 | -0.000 | -0.746 | 0.000 | 0.746 | 0.508 |
| | 3 | 2.537 | -0.214 | -0.057 | -0.028 | 0.223 | 0.612 |
| | 3 | 2.537 | 0.214 | -0.057 | 0.028 | 0.223 | 0.612 |
| | 3 | 4.395 | -0.207 | -0.035 | 0.259 | 0.334 | 0.612 |
| | 3 | 4.395 | 0.207 | -0.035 | -0.259 | 0.334 | 0.612 |
| | 4 | 1.519 | 0.113 | -0.000 | -0.079 | 0.138 | 0.707 |
| | 4 | 1.519 | -0.113 | 0.000 | 0.079 | 0.138 | 0.707 |
| | 1 | 1.168 | 0.070 | -0.215 | -0.058 | 0.233 | 0.832 |
| | 1 | 1.168 | -0.070 | -0.215 | 0.058 | 0.233 | 0.832 |
| | 2 | -2.272 | 0.039 | -0.074 | 0.058 | 0.102 | 0.871 |
| | 2 | -2.272 | -0.039 | -0.074 | -0.058 | 0.102 | 0.871 |
| | 2 | 0.140 | 0.070 | -0.117 | -0.081 | 0.158 | 0.871 |
| | 2 | 0.140 | -0.070 | -0.117 | 0.081 | 0.158 | 0.871 |
| | 4 | 1.745 | -0.000 | 0.026 | -0.000 | 0.026 | 1.000 |
| | 4 | 1.745 | 0.000 | -0.026 | 0.000 | 0.026 | 1.000 |
| Inter-layer interactions | 8 | 38.832 | 0.000 | -0.805 | -0.000 | 0.805 | 0.323 |
| | 5 | 17.941 | 0.000 | 0.506 | 0.000 | 0.506 | 0.358 |
| | 6 | 18.776 | -0.033 | 0.284 | 0.481 | 0.560 | 0.411 |
| | 6 | 18.776 | 0.033 | 0.284 | -0.481 | 0.560 | 0.411 |
| | 7 | 3.353 | -0.490 | -0.003 | 0.291 | 0.570 | 0.693 |
| | 7 | -2.174 | 0.124 | 0.077 | 0.289 | 0.324 | 0.693 |
| | 7 | -2.174 | -0.124 | 0.077 | -0.289 | 0.324 | 0.693 |
| | 7 | 3.353 | 0.490 | -0.003 | -0.291 | 0.570 | 0.693 |
| | 8 | -0.291 | 0.147 | -0.019 | -0.011 | 0.149 | 0.778 |
| | 8 | -0.291 | -0.147 | -0.019 | 0.011 | 0.149 | 0.778 |
| | 5 | -5.752 | -0.000 | 0.145 | 0.000 | 0.145 | 0.793 |
| | 5 | -0.857 | -0.010 | -0.128 | -0.087 | 0.155 | 0.793 |
| | 5 | -0.857 | 0.010 | -0.128 | 0.087 | 0.155 | 0.793 |
| | 8 | -2.708 | -0.000 | 0.115 | -0.000 | 0.115 | 1.051 |
| | 6 | 0.249 | 0.044 | -0.131 | -0.101 | 0.171 | 1.081 |
| | 6 | 0.249 | -0.044 | -0.131 | 0.101 | 0.171 | 1.081 |

**Table S5 | Heisenberg exchange interaction and DMI parameters for Fe 4 atom.** Both intra- and inter-layer interactions are taken into account. The distance between the neighbors is given in the units of the experimental lattice constant of InSb, 6.479 Å.

| Interactions for atom: Fe 5 | | | | | | | |
|---|---|---|---|---|---|---|---|
| | Neighbor atom (Fe) | $J_{ij}$ (meV) | $D_x$ (meV) | $D_y$ (meV) | $D_z$ (meV) | $\|\mathbf{D}\|$ (meV) | $\|\mathbf{R}\|$ (a) |
| Intra-layer interactions | 6 | 10.630 | 0.096 | -0.184 | -0.090 | 0.226 | 0.436 |
| | 6 | 10.630 | -0.096 | -0.184 | 0.090 | 0.226 | 0.436 |
| | 7 | 10.772 | 0.791 | 0.470 | -0.617 | 1.108 | 0.438 |
| | 7 | 10.772 | -0.791 | 0.470 | 0.617 | 1.108 | 0.438 |
| | 8 | 28.757 | 0.000 | -0.163 | -0.000 | 0.163 | 0.504 |
| | 8 | -4.314 | -0.000 | 0.100 | 0.000 | 0.100 | 0.504 |
| | 5 | -0.834 | -0.038 | -0.000 | 0.208 | 0.211 | 0.707 |
| | 5 | -0.834 | 0.038 | 0.000 | -0.208 | 0.211 | 0.707 |
| | 6 | -0.085 | -0.122 | 0.185 | -0.094 | 0.241 | 0.830 |
| | 6 | -0.085 | 0.122 | 0.185 | 0.094 | 0.241 | 0.830 |
| | 7 | -0.702 | 0.018 | -0.014 | -0.141 | 0.143 | 0.832 |
| | 7 | -0.702 | -0.018 | -0.014 | 0.141 | 0.143 | 0.832 |
| | 8 | -0.820 | 0.029 | -0.043 | -0.106 | 0.118 | 0.869 |
| | 8 | -0.820 | -0.029 | -0.043 | 0.106 | 0.118 | 0.869 |
| | 8 | -1.249 | 0.075 | -0.078 | -0.062 | 0.125 | 0.869 |
| | 8 | -1.249 | -0.075 | -0.078 | 0.062 | 0.125 | 0.869 |
| | 5 | -3.234 | 0.000 | -0.056 | 0.000 | 0.056 | 1.000 |
| | 5 | -3.234 | -0.000 | 0.056 | -0.000 | 0.056 | 1.000 |
| Inter-layer interactions | 2 | 16.590 | -0.000 | -0.131 | 0.000 | 0.131 | 0.300 |
| | 4 | 17.941 | -0.000 | -0.506 | -0.000 | 0.506 | 0.358 |
| | 3 | 12.908 | -0.166 | 0.016 | 0.044 | 0.172 | 0.508 |
| | 3 | 12.908 | 0.166 | 0.016 | -0.044 | 0.172 | 0.508 |
| | 1 | -6.111 | 0.186 | 0.071 | 0.042 | 0.204 | 0.639 |
| | 1 | -4.370 | -0.124 | -0.119 | 0.163 | 0.237 | 0.639 |
| | 1 | -4.370 | 0.124 | -0.119 | -0.163 | 0.237 | 0.639 |
| | 1 | -6.111 | -0.186 | 0.071 | -0.042 | 0.204 | 0.639 |
| | 2 | 0.688 | -0.040 | 0.015 | 0.030 | 0.052 | 0.768 |
| | 2 | 0.688 | 0.040 | 0.015 | -0.030 | 0.052 | 0.768 |
| | 2 | -9.858 | -0.000 | 0.265 | 0.000 | 0.265 | 0.768 |
| | 4 | -0.857 | 0.010 | 0.128 | 0.087 | 0.155 | 0.793 |
| | 4 | -0.857 | -0.010 | 0.128 | -0.087 | 0.155 | 0.793 |
| | 4 | -5.752 | 0.000 | -0.145 | -0.000 | 0.145 | 0.793 |
| | 3 | -1.394 | 0.030 | 0.021 | -0.102 | 0.109 | 0.871 |
| | 3 | -1.394 | -0.030 | 0.021 | 0.102 | 0.109 | 0.871 |
| | 2 | -0.484 | -0.013 | -0.097 | -0.039 | 0.105 | 1.044 |
| | 2 | -0.484 | 0.013 | -0.097 | 0.039 | 0.105 | 1.044 |

**Table S6 | Heisenberg exchange interaction and DMI parameters for Fe 5 atom.** Both intra- and inter-layer interactions are taken into account. The distance between the neighbors is given in the units of the experimental lattice constant of InSb, 6.479 Å.

| Interactions for atom: Fe 6 | | | | | | | |
|---|---|---|---|---|---|---|---|
| | Neighbor atom (Fe) | $J_{ij}$ (meV) | $D_x$ (meV) | $D_y$ (meV) | $D_z$ (meV) | $\|\mathbf{D}\|$ (meV) | $\|\mathbf{R}\|$ (a) |
| Intra-layer interactions | 5 | 10.630 | 0.096 | 0.184 | -0.090 | 0.226 | 0.436 |
| | 5 | 10.630 | -0.096 | 0.184 | 0.090 | 0.226 | 0.436 |
| | 8 | 11.922 | 0.099 | -0.340 | 0.030 | 0.355 | 0.448 |
| | 8 | 11.922 | -0.099 | -0.340 | -0.030 | 0.355 | 0.448 |
| | 7 | 18.670 | 0.000 | 0.163 | -0.000 | 0.163 | 0.513 |
| | 7 | -5.512 | 0.000 | -0.104 | 0.000 | 0.104 | 0.513 |
| | 6 | -4.925 | 0.193 | -0.000 | 0.196 | 0.275 | 0.707 |
| | 6 | -4.925 | -0.193 | 0.000 | -0.196 | 0.275 | 0.707 |
| | 5 | -0.085 | 0.122 | -0.185 | 0.094 | 0.241 | 0.830 |
| | 5 | -0.085 | -0.122 | -0.185 | -0.094 | 0.241 | 0.830 |
| | 8 | 0.513 | -0.039 | -0.152 | -0.327 | 0.363 | 0.837 |
| | 8 | 0.513 | 0.039 | -0.152 | 0.327 | 0.363 | 0.837 |
| | 7 | -3.173 | 0.026 | 0.092 | 0.213 | 0.234 | 0.873 |
| | 7 | -3.173 | -0.026 | 0.092 | -0.213 | 0.234 | 0.873 |
| | 7 | -0.553 | 0.009 | 0.028 | 0.032 | 0.043 | 0.873 |
| | 7 | -0.553 | -0.009 | 0.028 | -0.032 | 0.043 | 0.873 |
| | 6 | -0.560 | -0.000 | -0.126 | -0.000 | 0.126 | 1.000 |
| | 6 | -0.560 | 0.000 | 0.126 | 0.000 | 0.126 | 1.000 |
| Inter-layer interactions | 1 | 9.022 | 0.000 | 0.323 | 0.000 | 0.323 | 0.284 |
| | 3 | 17.500 | 0.000 | 0.438 | -0.000 | 0.438 | 0.333 |
| | 4 | 18.776 | 0.033 | -0.284 | -0.481 | 0.560 | 0.411 |
| | 4 | 18.776 | -0.033 | -0.284 | 0.481 | 0.560 | 0.411 |
| | 2 | -0.438 | -0.044 | 0.027 | 0.083 | 0.098 | 0.624 |
| | 2 | -0.438 | 0.044 | 0.027 | -0.083 | 0.098 | 0.624 |
| | 2 | 0.354 | 0.111 | 0.298 | -0.056 | 0.323 | 0.624 |
| | 2 | 0.354 | -0.111 | 0.298 | 0.056 | 0.323 | 0.624 |
| | 1 | 0.202 | -0.029 | -0.047 | -0.098 | 0.113 | 0.762 |
| | 1 | 0.202 | 0.029 | -0.047 | 0.098 | 0.113 | 0.762 |
| | 1 | -9.165 | 0.000 | 0.016 | -0.000 | 0.016 | 0.762 |
| | 3 | 0.874 | -0.043 | -0.010 | 0.060 | 0.074 | 0.782 |
| | 3 | 0.874 | 0.043 | -0.010 | -0.060 | 0.074 | 0.782 |
| | 3 | 2.375 | 0.000 | 0.006 | 0.000 | 0.006 | 0.782 |
| | 1 | -0.878 | -0.013 | -0.100 | -0.127 | 0.163 | 1.040 |
| | 1 | -0.878 | 0.013 | -0.100 | 0.127 | 0.163 | 1.040 |
| | 4 | 0.249 | 0.044 | 0.131 | -0.101 | 0.171 | 1.081 |
| | 4 | 0.249 | -0.044 | 0.131 | 0.101 | 0.171 | 1.081 |

**Table S7 | Heisenberg exchange interaction and DMI parameters for Fe 6 atom.** Both intra- and inter-layer interactions are taken into account. The distance between the neighbors is given in the units of the experimental lattice constant of InSb, 6.479 Å.

| Interactions for atom: Fe 7 | | | | | | | |
|---|---|---|---|---|---|---|---|
| | Neighbor atom (Fe) | $J_{ij}$ (meV) | $D_x$ (meV) | $D_y$ (meV) | $D_z$ (meV) | $\lvert\mathbf{D}\rvert$ (meV) | $\lvert\mathbf{R}\rvert$ (a) |
| Intra-layer interactions | 5 | 10.772 | 0.791 | -0.470 | -0.617 | 1.108 | 0.438 |
| | 5 | 10.772 | -0.791 | -0.470 | 0.617 | 1.108 | 0.438 |
| | 6 | 18.670 | -0.000 | -0.163 | 0.000 | 0.163 | 0.513 |
| | 6 | -5.512 | -0.000 | 0.104 | -0.000 | 0.104 | 0.513 |
| | 8 | -0.472 | 0.020 | 0.142 | -0.018 | 0.144 | 0.612 |
| | 8 | 0.059 | 0.512 | 0.536 | 0.308 | 0.802 | 0.612 |
| | 8 | -0.472 | -0.020 | 0.142 | 0.018 | 0.144 | 0.612 |
| | 8 | 0.059 | -0.512 | 0.536 | -0.308 | 0.802 | 0.612 |
| | 7 | 1.005 | -0.214 | -0.000 | 0.221 | 0.307 | 0.707 |
| | 7 | 1.005 | 0.214 | 0.000 | -0.221 | 0.307 | 0.707 |
| | 5 | -0.702 | -0.018 | 0.014 | 0.141 | 0.143 | 0.832 |
| | 5 | -0.702 | 0.018 | 0.014 | -0.141 | 0.143 | 0.832 |
| | 6 | -0.553 | 0.009 | -0.028 | 0.032 | 0.043 | 0.873 |
| | 6 | -0.553 | -0.009 | -0.028 | -0.032 | 0.043 | 0.873 |
| | 6 | -3.173 | 0.026 | -0.092 | 0.213 | 0.234 | 0.873 |
| | 6 | -3.173 | -0.026 | -0.092 | -0.213 | 0.234 | 0.873 |
| | 7 | 0.571 | 0.000 | 0.018 | -0.000 | 0.018 | 1.000 |
| | 7 | 0.571 | -0.000 | -0.018 | 0.000 | 0.018 | 1.000 |
| Inter-layer interactions | 3 | 44.387 | -0.000 | -0.384 | 0.000 | 0.384 | 0.334 |
| | 1 | 29.892 | 0.000 | -0.628 | -0.000 | 0.628 | 0.353 |
| | 2 | 10.437 | 0.248 | -0.037 | 0.037 | 0.253 | 0.424 |
| | 2 | 10.437 | -0.248 | -0.037 | -0.037 | 0.253 | 0.424 |
| | 4 | -2.174 | 0.124 | -0.077 | 0.289 | 0.324 | 0.693 |
| | 4 | -2.174 | -0.124 | -0.077 | -0.289 | 0.324 | 0.693 |
| | 4 | 3.353 | 0.490 | 0.003 | -0.291 | 0.570 | 0.693 |
| | 4 | 3.353 | -0.490 | 0.003 | 0.291 | 0.570 | 0.693 |
| | 3 | 0.930 | 0.203 | -0.002 | -0.015 | 0.203 | 0.782 |
| | 3 | 0.930 | -0.203 | -0.002 | 0.015 | 0.203 | 0.782 |
| | 1 | -4.700 | 0.000 | 0.033 | 0.000 | 0.033 | 0.790 |
| | 1 | 0.378 | -0.204 | -0.185 | -0.346 | 0.442 | 0.790 |
| | 1 | 0.378 | 0.204 | -0.185 | 0.346 | 0.442 | 0.790 |
| | 3 | 2.023 | -0.000 | 0.009 | 0.000 | 0.009 | 1.054 |
| | 2 | 0.486 | 0.019 | -0.086 | -0.113 | 0.144 | 1.086 |
| | 2 | 0.486 | -0.019 | -0.086 | 0.113 | 0.144 | 1.086 |

**Table S8 | Heisenberg exchange interaction and DMI parameters for Fe 7 atom.** Both intra- and inter-layer interactions are taken into account. The distance between the neighbors is given in the units of the experimental experimental lattice constant of InSb, 6.479 Å.

| Interactions for atom: Fe 8 | | | | | | | |
|---|---|---|---|---|---|---|---|
| | Neighbor atom (Fe) | $J_{ij}$ (meV) | $D_x$ (meV) | $D_y$ (meV) | $D_z$ (meV) | $\|\mathbf{D}\|$ (meV) | $\|\mathbf{R}\|$ (a) |
| Intra-layer interactions | 6 | 11.922 | -0.099 | 0.340 | -0.030 | 0.355 | 0.448 |
| | 6 | 11.922 | 0.099 | 0.340 | 0.030 | 0.355 | 0.448 |
| | 5 | -4.314 | 0.000 | -0.100 | -0.000 | 0.100 | 0.504 |
| | 5 | 28.757 | -0.000 | 0.163 | 0.000 | 0.163 | 0.504 |
| | 7 | 0.059 | -0.512 | -0.536 | -0.308 | 0.802 | 0.612 |
| | 7 | 0.059 | 0.512 | -0.536 | 0.308 | 0.802 | 0.612 |
| | 7 | -0.472 | 0.020 | -0.142 | -0.018 | 0.144 | 0.612 |
| | 7 | -0.472 | -0.020 | -0.142 | 0.018 | 0.144 | 0.612 |
| | 8 | -4.057 | -0.451 | -0.000 | 0.013 | 0.452 | 0.707 |
| | 8 | -4.057 | 0.451 | 0.000 | -0.013 | 0.452 | 0.707 |
| | 6 | 0.513 | -0.039 | 0.152 | -0.327 | 0.363 | 0.837 |
| | 6 | 0.513 | 0.039 | 0.152 | 0.327 | 0.363 | 0.837 |
| | 5 | -1.249 | -0.075 | 0.078 | 0.062 | 0.125 | 0.869 |
| | 5 | -1.249 | 0.075 | 0.078 | -0.062 | 0.125 | 0.869 |
| | 5 | -0.820 | -0.029 | 0.043 | 0.106 | 0.118 | 0.869 |
| | 5 | -0.820 | 0.029 | 0.043 | -0.106 | 0.118 | 0.869 |
| | 8 | -1.024 | 0.000 | 0.019 | 0.000 | 0.019 | 1.000 |
| | 8 | -1.024 | -0.000 | -0.019 | -0.000 | 0.019 | 1.000 |
| Inter-layer interactions | 4 | 38.832 | -0.000 | 0.805 | 0.000 | 0.805 | 0.323 |
| | 2 | 39.792 | 0.000 | -0.408 | -0.000 | 0.408 | 0.342 |
| | 1 | 8.547 | 0.067 | -0.349 | 0.345 | 0.496 | 0.433 |
| | 1 | 8.547 | -0.067 | -0.349 | -0.345 | 0.496 | 0.433 |
| | 3 | -2.512 | 0.033 | 0.118 | -0.148 | 0.192 | 0.697 |
| | 3 | -2.512 | -0.033 | 0.118 | 0.148 | 0.192 | 0.697 |
| | 3 | 2.947 | 0.023 | -0.158 | -0.053 | 0.168 | 0.697 |
| | 3 | 2.947 | -0.023 | -0.158 | 0.053 | 0.168 | 0.697 |
| | 4 | -0.291 | 0.147 | 0.019 | -0.011 | 0.149 | 0.778 |
| | 4 | -0.291 | -0.147 | 0.019 | 0.011 | 0.149 | 0.778 |
| | 2 | -6.573 | -0.000 | -0.109 | -0.000 | 0.109 | 0.785 |
| | 2 | -0.703 | -0.185 | 0.104 | -0.012 | 0.213 | 0.785 |
| | 2 | -0.703 | 0.185 | 0.104 | 0.012 | 0.213 | 0.785 |
| | 4 | -2.708 | 0.000 | -0.115 | 0.000 | 0.115 | 1.051 |
| | 1 | 0.438 | -0.124 | -0.076 | 0.163 | 0.218 | 1.090 |
| | 1 | 0.438 | 0.124 | -0.076 | -0.163 | 0.218 | 1.090 |

**Table S9 | Heisenberg exchange interaction and DMI parameters for Fe 8 atom.** Both intra- and inter-layer interactions are taken into account. The distance between the neighbors is given in the units of the experimental lattice constant of InSb, 6.479 Å.

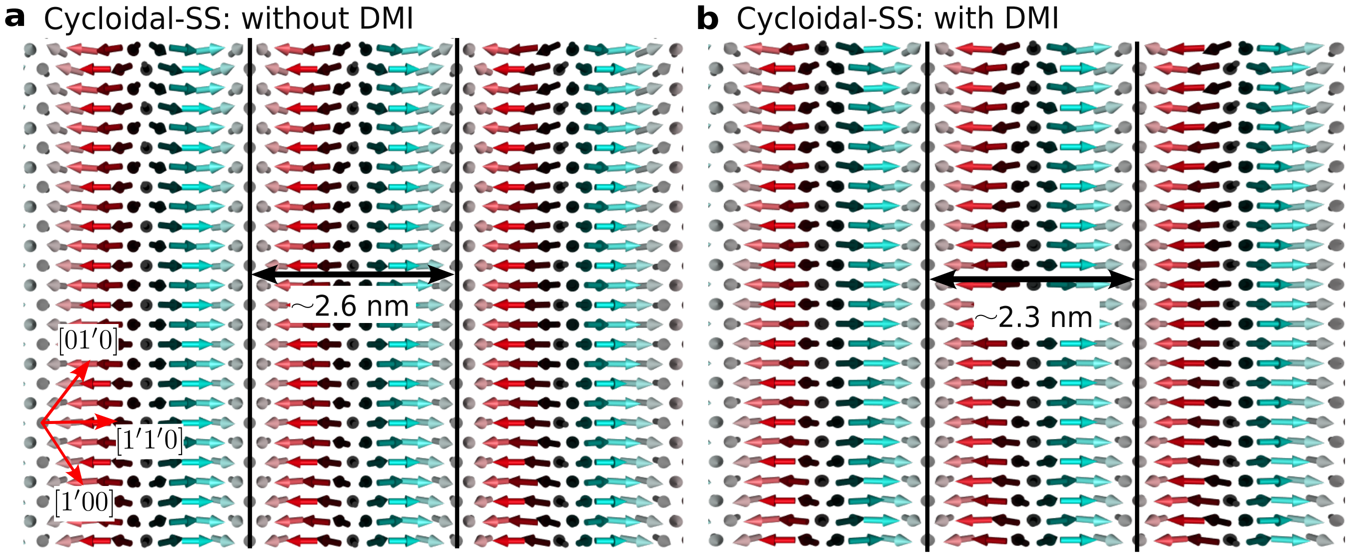


**Fig. S6 | Emergent spin spiral states: frustrated vs. chiral. a,** A spontaneous SS solution in the absence of DMI and magnetocrystalline anisotropy. **b,** A cycloidal-SS structure with DMI and magnetocrystalline anisotropy. Ultimately, the ground state in the absence of a magnetic field is a left-handed cycloidal-SS characterized by an atomic-scale period. To enhance visual clarity, only the top Fe layer is illustrated in these figures.

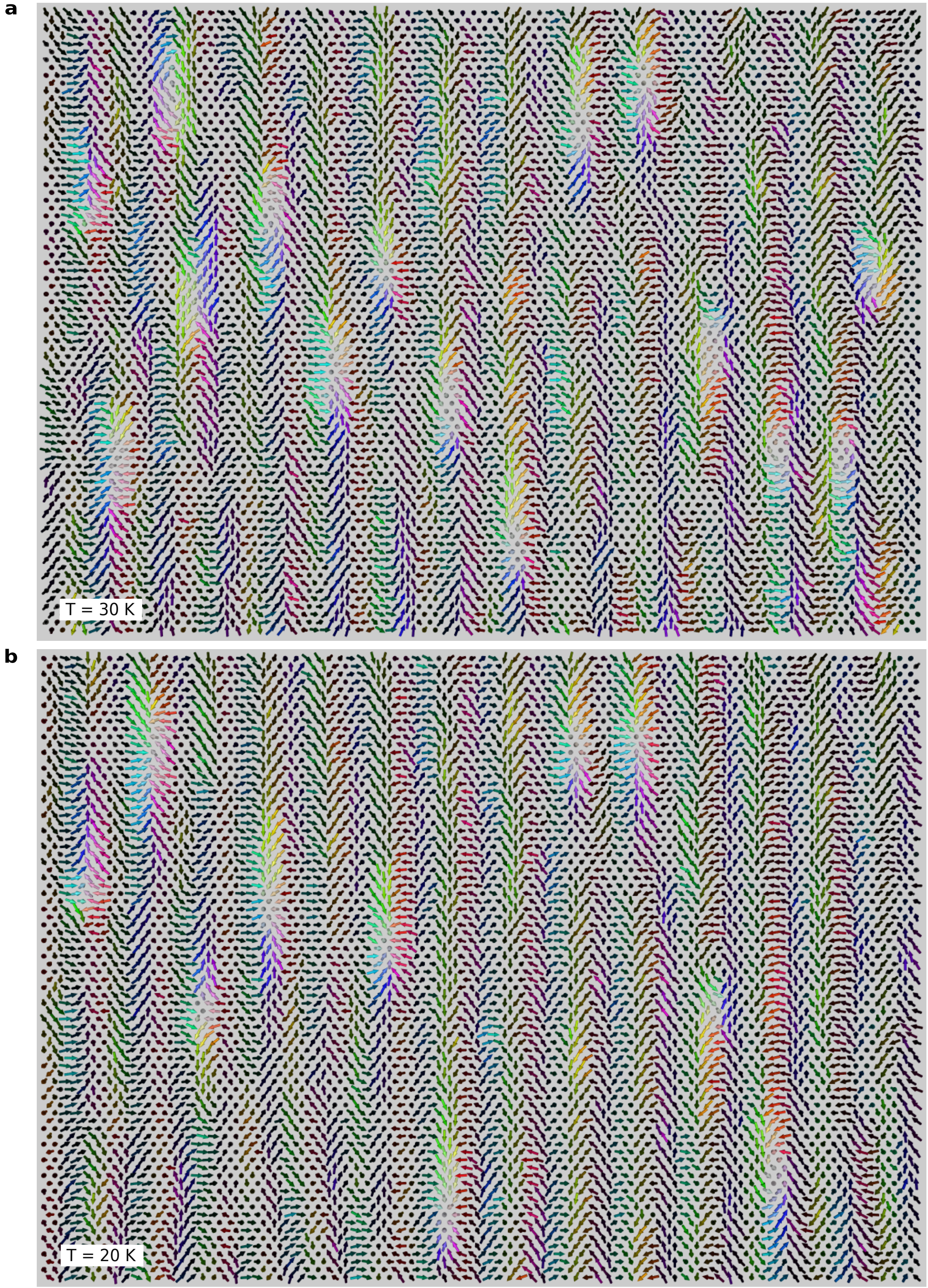
a
T = 30 K
b
T = 20 K

c

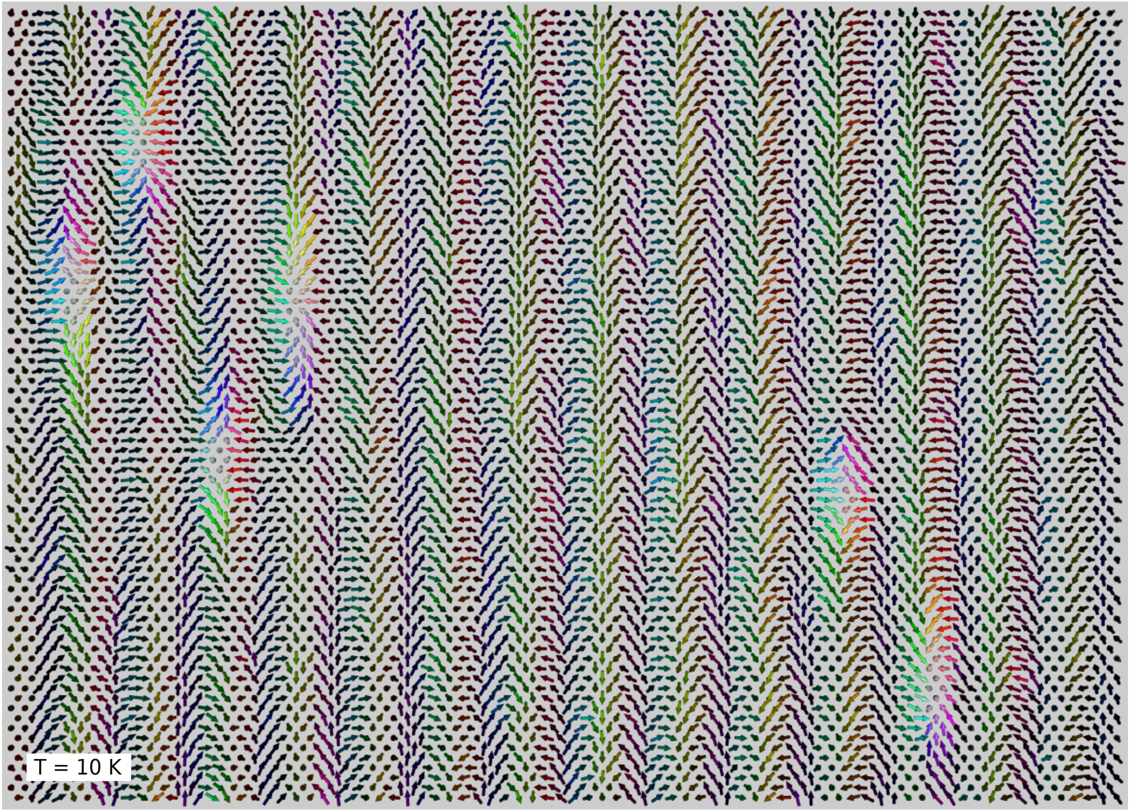


d

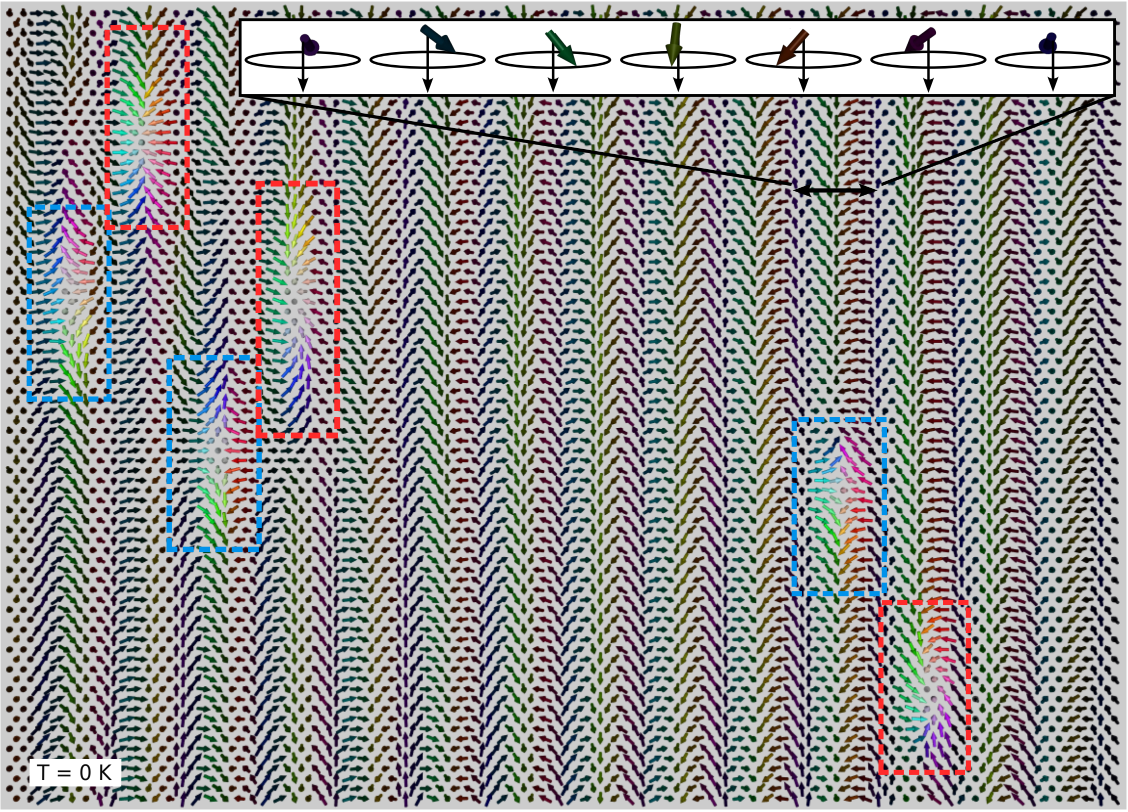


**Fig. S7 | Magnetic field induced spontaneous nucleation of both skyrmions and antiskyrmions in a finite domain.** At finite temperature about 50 K, simulations are initiated with a random distribution of spin orientations across the lattice sites. The temperature is systematically reduced in 0.5 K steps. **a-d**, the spin configurations obtained from our simulations at temperatures, 30 K, 20 K, 10 K, and 0 K, respectively. In **d**, skyrmions are highlighted with red boxes, while antiskyrmions are marked with blue boxes. The inset provides a magnified view of the underlying cone-SS background modulation.

a

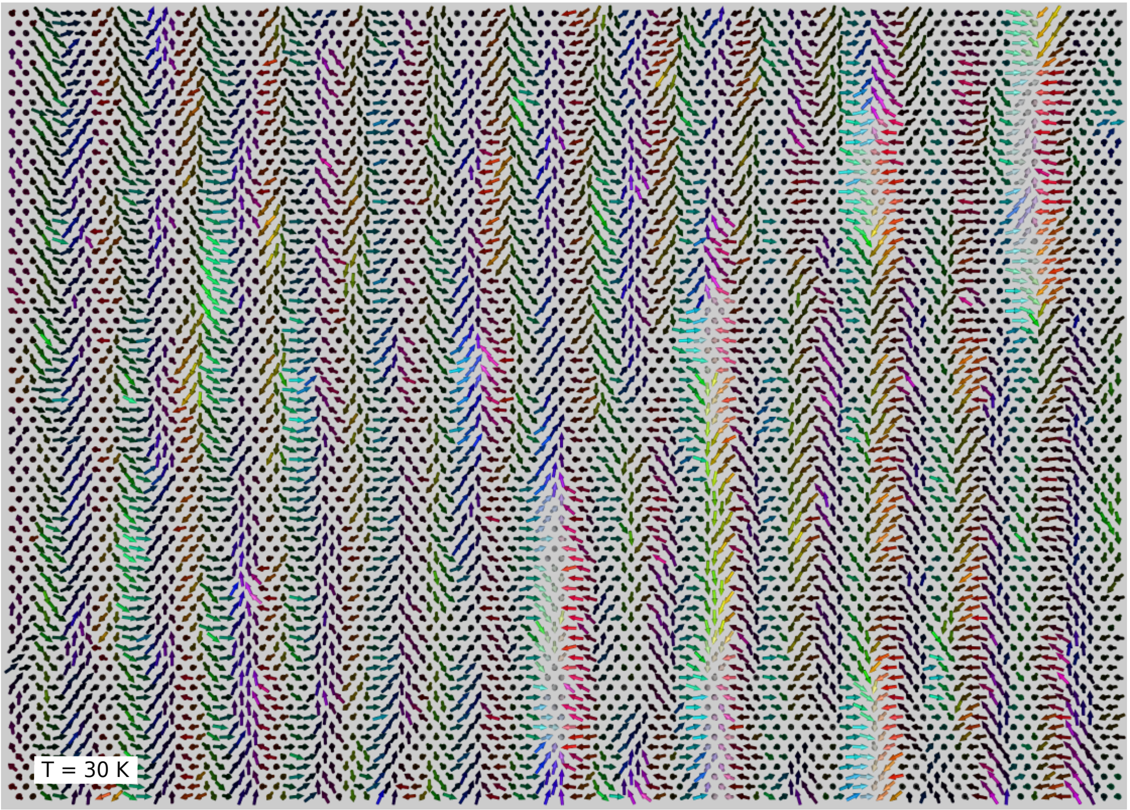


b

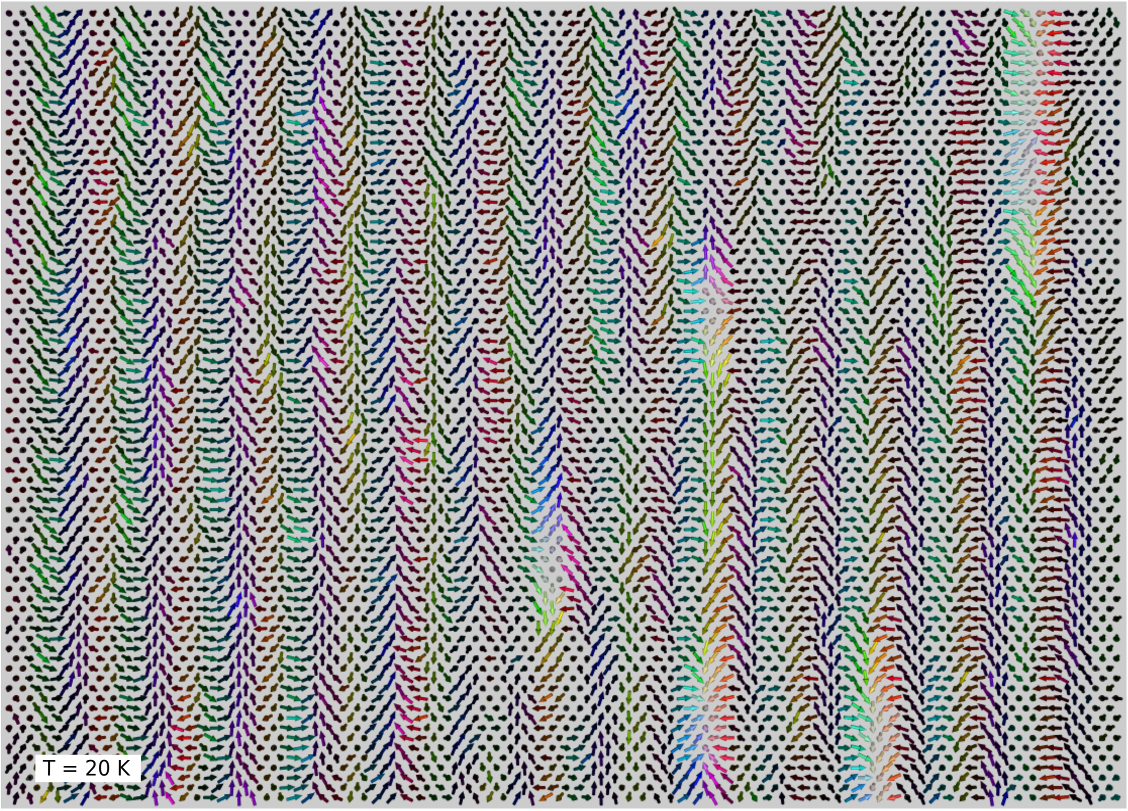

c

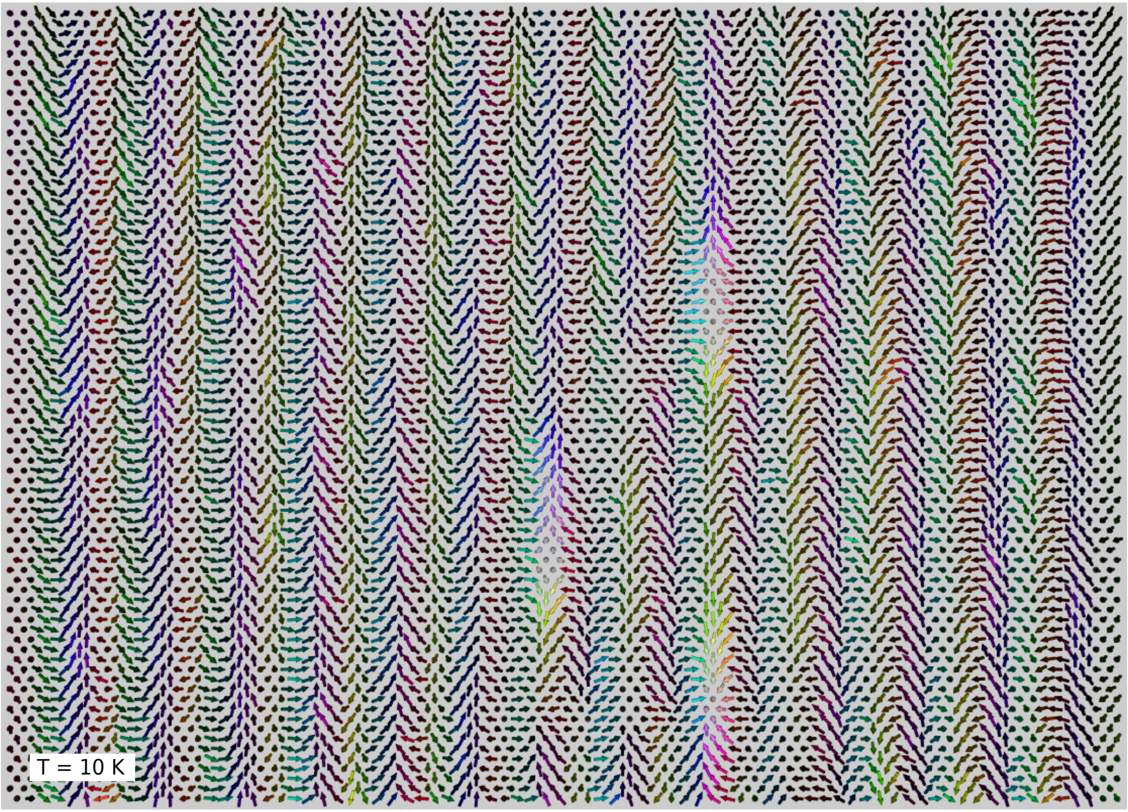


d

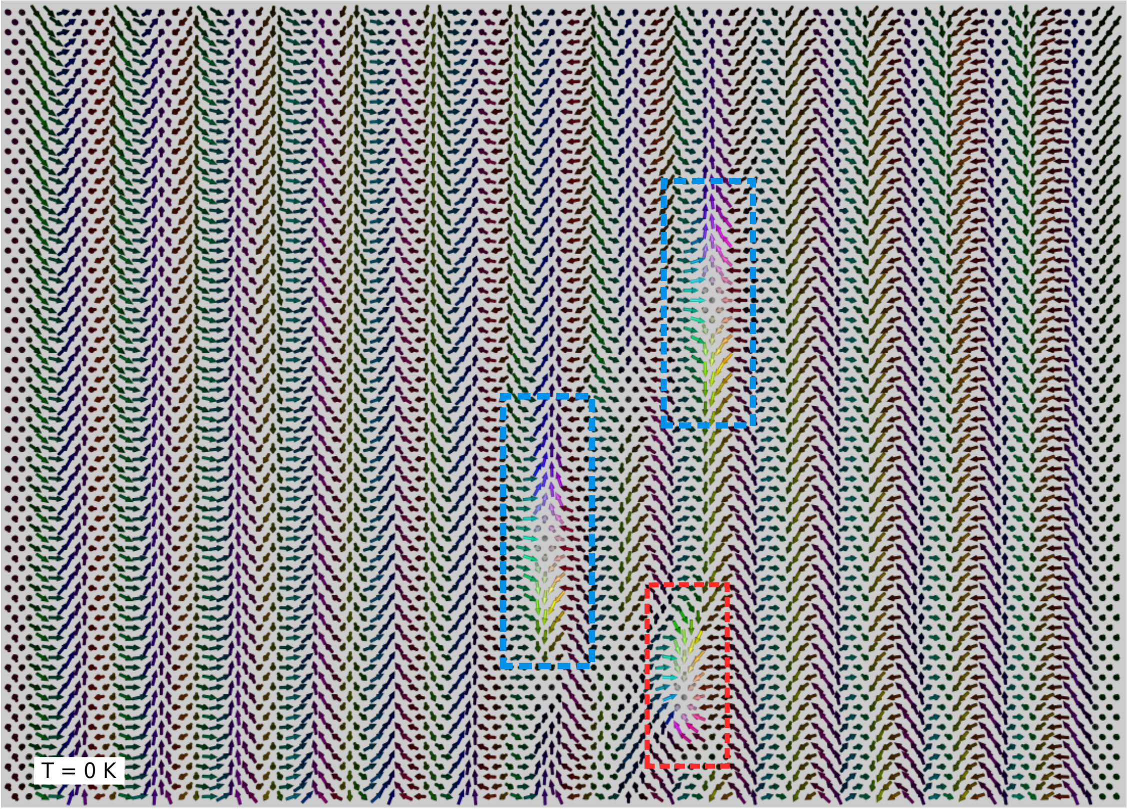


**Fig. S8 | Magnetic field induced spontaneous nucleation of both skyrmions and antiskyrmions in a finite domain initialize with cycloidal-SS state.** Following a similar procedure as in previous studies, but instead of employing a random spin configuration as the initial state, we initiate the simulations with the ground state cycloidal-SS configuration. **a-d**, the spin configurations obtained from our simulations at temperatures of 30 K, 20 K, 10 K, and 0 K, respectively. In **d**, skyrmions and antiskyrmions are highlighted with red and blue boxes, respectively.

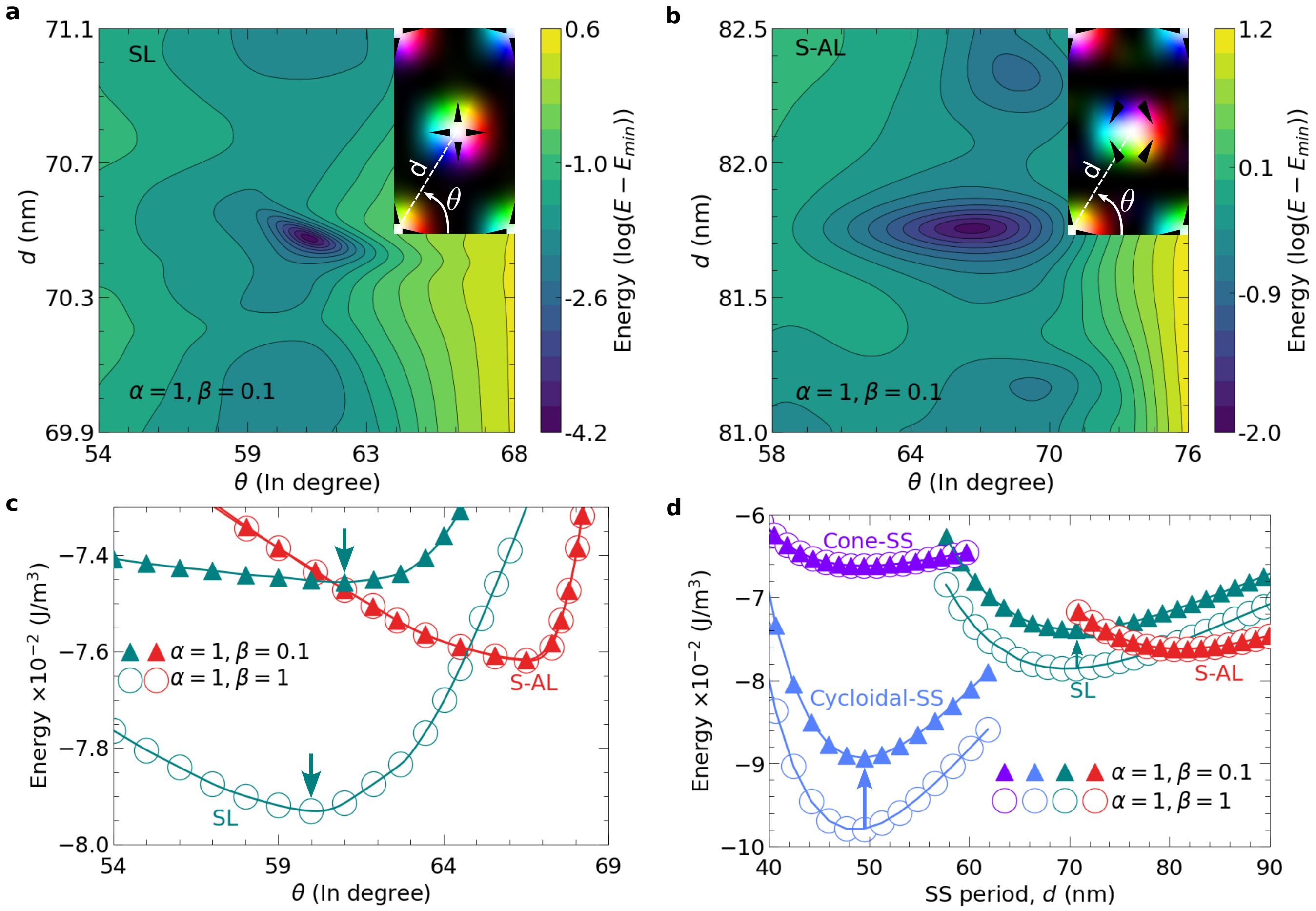


**Fig. S9 | Energy optimization of noncollinear magnetic phases:** Contour color maps in panels **a** and **b** depict the energy density (per unit volume) of the SL and S-AL, respectively, as a function of the *core-to-core* distance, $d$, and shape parameter, $\theta$. The inset illustrates the definition of these parameters within a rectangular unit cell. All energy points are calculated relative to the minimum energy configuration, $E_{\min}$, and presented on a logarithmic scale. The corresponding color variation is shown on the right. Anisotropy in DMI magnitudes is considered, with $\beta$ fixed at 0.1. **c,** Energy profile of the unit cell as a function of the shape parameter $\theta$, obtained through direct energy minimization. **d,** Energy density as a function of the *core-to-core* distance $d$ within the SL and S-AL unit cells, and as a function of the period for the cone-SS and cycloidal-SS phases. The energy minima correspond to the equilibrium configurations. Here, we have maintained a constant external magnetic field, $h = 0.35$. The optimized energy density for both S-AL and cone-SS phases remains invariant with respect to variations in the anisotropy parameter $\beta$. However, upon introducing anisotropy in the DMI magnitude ($\beta = 0.1$), we observe a significant increase in the energy density profiles of both the cycloidal-SS and SL phases compared to the isotropic case ($\beta = 1$). Despite the presence of DMI anisotropy, it is noteworthy that both optimized lattice states remain metastable regardless of the applied field. As $\beta = 0.1 (< \beta_c)$, S-AL becomes the energetically favorable phase compared to the SL. The optimized lattices for both phases are shown in the main text Figs. **2a** and **b** for SL and S-AL, respectively.

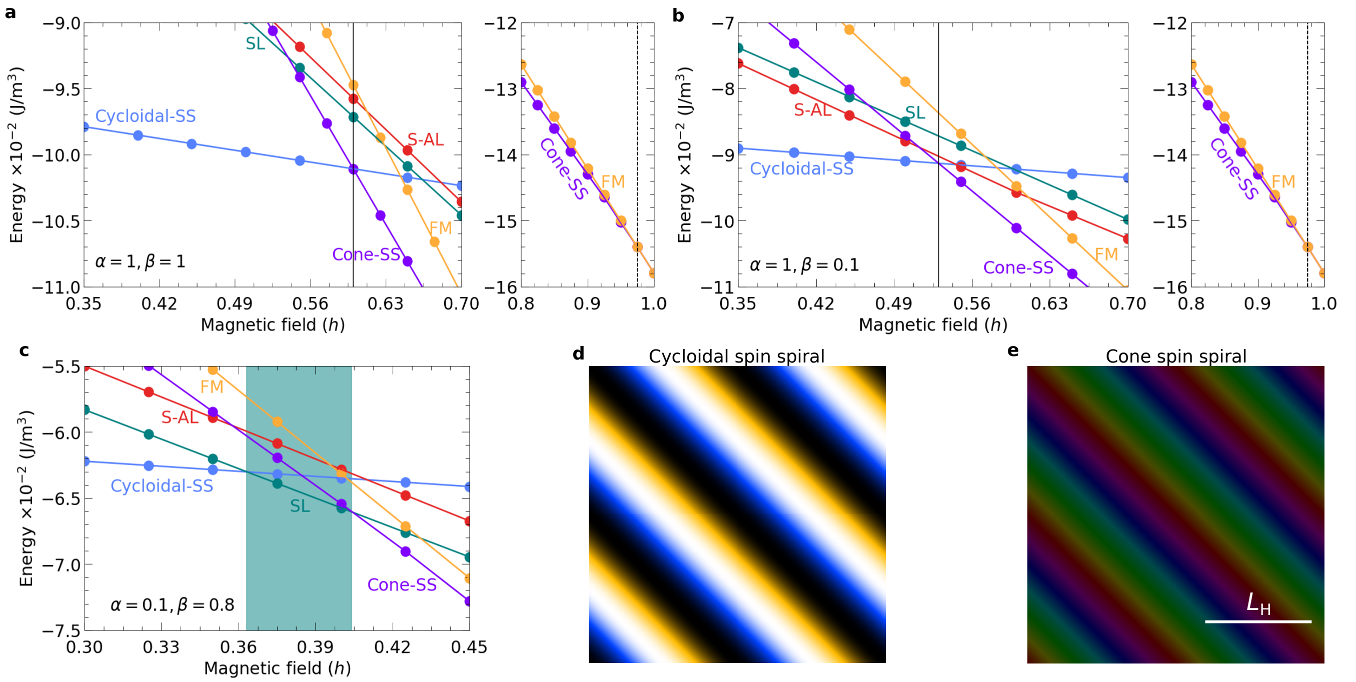


**Fig. S10 | Energy density vs. magnetic field of competing non-collinear magnetic phases:** This figure further extends our analysis by exploring the critical behavior of the anisotropy parameter $\beta$. This significantly influences the energy lines and the stability of various magnetic phases. **a,** Pure isotropic case. The energy densities shown in this figure are calculated using a simplified model of a frustrated chiral magnet, which omits the influence of magnetocrystalline anisotropy. The cycloidal-SS state remains the ground state for applied fields up to approximately 0.6. Beyond this critical field, the cone-SS emerges as the energetically favorable state. As depicted in the rightmost figure, a second-order phase transition occurs from the cone-SS to the saturated FM phase at high magnetic fields, indicated by the vertical line. While both the SL and S-AL phases are metastable, the SL energy is always lower than that of S-AL. **b,** A case with anisotropy in DMI below the critical value, $\beta_c \sim 0.55$ (see Fig. **S11c**). While the overall phase transition sequence remains consistent with the isotropic case, the introduction of anisotropy ($\beta = 0.1$) leads to a subtle shift in energy within the lattice phases, favoring the S-AL over the SL. **c,** A scenario with both anisotropic interactions, DMI and exchange, *i.e.*, $\beta = 0.8$ ($> \beta_c$) and $\alpha = 0.1$. In contrast to Fig. **2e**, first-order phase transitions from the cycloidal-SS to the SL and subsequently from the SL to the cone-SS are observed at critical fields of approximately $h \approx 0.364$ and 0.405, respectively. Within the 2D domain, **d,** the cycloidal-SS and **e,** the cone-SS phase are determined through direct energy minimization of model (**2**). Also, Figs. **1f** and **g** illustrate the characteristic spin orientations of the cycloidal-SS and cone-SS phases, respectively.

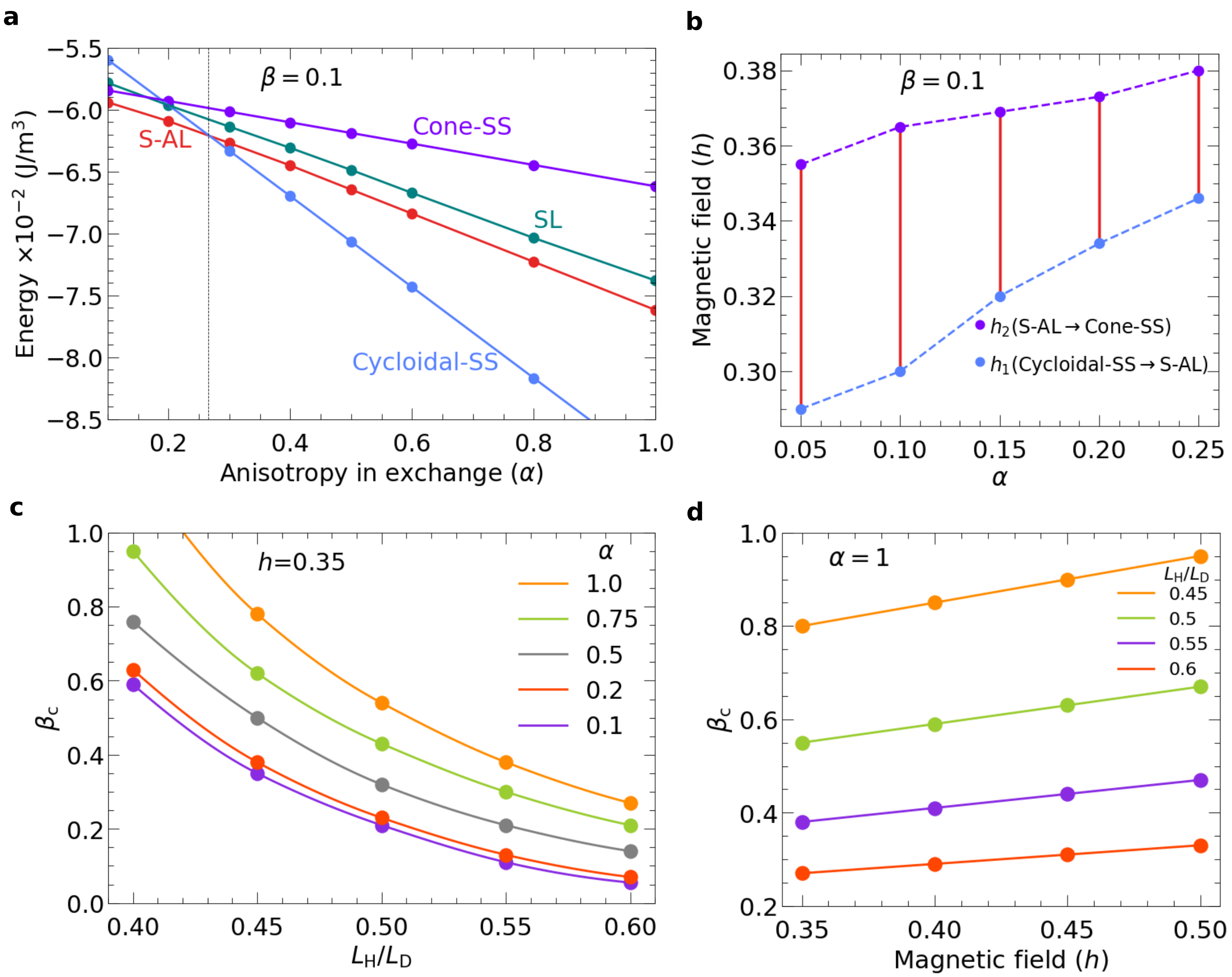


**Fig. S11 | The role of $\alpha$ and $L_\mathrm{H}/L_\mathrm{D}$ in stabilizing S-AL phase: a,** Energy comparison of competing non-collinear states (cycloidal-SS, cone-SS, SL, and S-AL) as a function of the exchange anisotropy parameter $\alpha$, with $\beta$ fixed at 0.1. The vertical line marks the critical value of $\alpha$, below which the S-AL phase becomes energetically favorable magnetic state. For this simulation, a constant external magnetic field of $h = 0.35$ is applied, while keeping the ratio of $L_\mathrm{H}/L_\mathrm{D}$ fixed at 0.5. **b,** Stability window of the ground-state S-AL phase as a function of the anisotropy parameter $\alpha$, below its critical value of 0.26. The magnetic fields $h_1$ and $h_2$ demarcate the phase boundaries between the cycloidal-SS, S-AL, and cone-SS phases. A notable correlation exists between $\alpha$ and the stability field window of the S-AL phase, with decreasing $\alpha$ resulting in an expanded window. **c,** Dependence of $\beta_\mathrm{c}$ for S-AL stability on various values of $L_\mathrm{H}/L_\mathrm{D}$ and $\alpha$. For values of $\beta$ below the critical value $\beta_\mathrm{c}$, the S-AL can be emerged as the energetically favored ground state within a specific range of external magnetic fields. This range is bounded by two critical field values that define the transitions between the cycloidal-SS, S-AL, and cone-SS phases. Note, with $L_\mathrm{H}/L_\mathrm{D} = 0.56$, $\beta_\mathrm{c}$ is approximately 0.09. Therefore, the S-AL phase is absent as the ground state in Fig. **3b** for all values of $h$. **d,** Dependence of the critical parameter $\beta_\mathrm{c}$, crucial for S-AL stability, on variations in $h$ for different $L_\mathrm{H}/L_\mathrm{D}$ ratios. Here, with $\alpha$ held constant at unity, we observed a linear relationship between $\beta_\mathrm{c}$ and $h$.

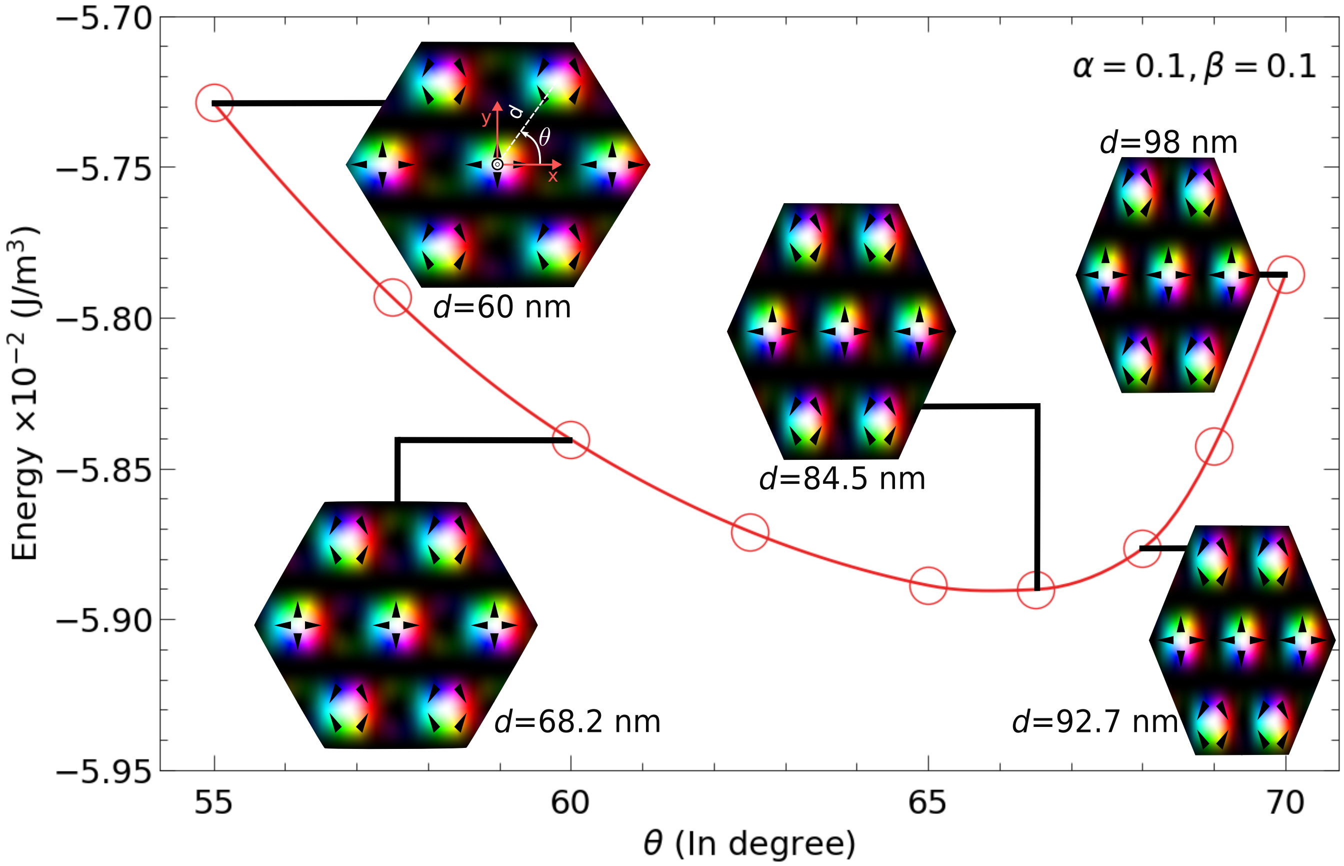


**Fig. S12 | Minimum energy S-AL with elongated skyrmion and antiskyrmion configurations:** Minimum energy density of the S-AL configuration determined by optimizing the *core-to-core* distance ($d$) within a rectangular unit cell for each value of the fixed angle (shape parameter) $\theta$. For the anisotropic system with $\alpha = \beta = 0.1$, the distance $d$ corresponds to the snapshot exhibiting minimum energy density. The equilibrium S-AL phase with the lowest energy is found at $\theta = 66.5°$, exhibiting elongated skyrmions and antiskyrmions as shown in Fig. **2c**. Here, our results unveil a significant correlation between the lattice shape and the skyrmion's elongation within the S-AL phase. Notably, the direction of elongation undergoes a marked change below $\theta = 60°$. For example, comparing S-AL configurations above and below this angle demonstrates a clear orthogonality in their elongation directions. This intriguing observation emphasizes the profound influence of DMI anisotropy on dictating the elongation direction. This also happens when we set $\alpha = 1$.

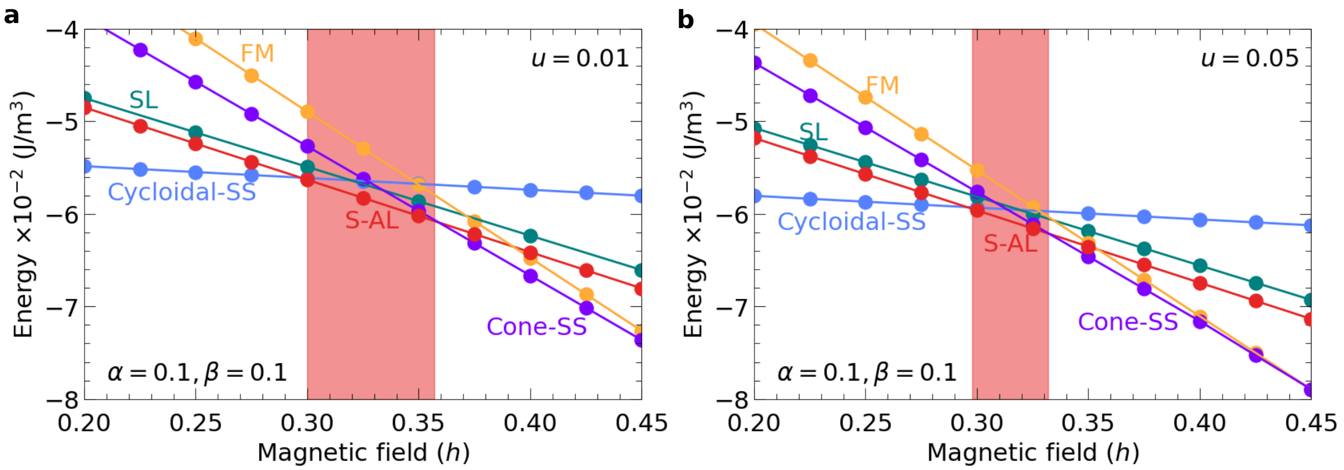


**Fig. S13 | Role of magnetocrystalline anisotropy parameter $u$:** By incorporating magnetocrystalline anisotropy as an additional energy term into our model (**2**) (see Methods), we demonstrate here a more comprehensive understanding of 2D chiral magnets. **a** and **b** illustrate the impact of easy-axis anisotropy ($u = 0.01$ and 0.05, respectively) on the stability of the S-AL phase. This extends the analysis presented in Fig. **2g** to the case of non-zero easy-axis anisotropy ($u \neq 0$). Importantly, easy-axis anisotropy favors the cone-SS and saturated FM phases, leading to a second-order phase transition at lower magnetic fields with increasing $u$. As a result, the stability range of the S-AL phase in terms of the external field decreases with increasing $u$.

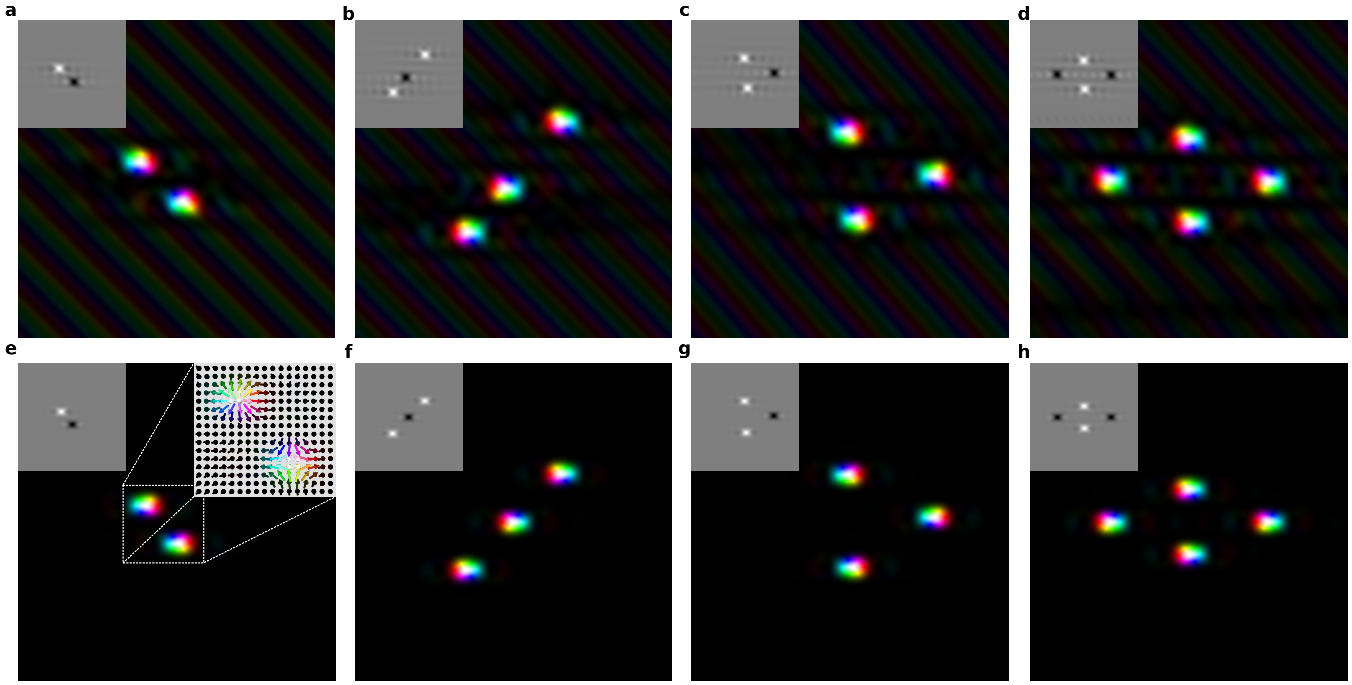


**Fig. S14 | Stable skyrmion-antiskyrmion clusters in anisotropic chiral magnet:** Micromagnetic simulations within our frustrated chiral magnet with $\alpha = \beta = 0.1$ reveal the formation of stable, multi-skyrmion clusters within a 2D domain. These clusters include: **a,** a skyrmion-antiskyrmion pair, **b,** linear and **c,** triangular arrangements of two skyrmions and one antiskyrmion, and **d,** a cluster of two skyrmions and two antiskyrmions. Starting from initial configurations topologically equivalent to the desired final skyrmion cluster configurations, we have performed direct energy minimization of our model under an external magnetic field, $h = 0.5$. This minimization process is continued until a stable, minimum-energy configuration is reached. All clusters appear to be embedded within the domain, which exhibits a cone-SS modulation in the background magnetization. Upon increasing the magnetic field to $h = 0.7$, the cone-SS background magnetization is suppressed, resulting in a homogeneous magnetization with skyrmion clusters unaltered, as shown in panels **e-h**. The insets in each figure depict the topological charge density distribution within the domain. Concentrated black (white) dots against the gray background signify the presence of antiskyrmions (skyrmions) with topological charge $Q = 1(-1)$, respectively. Upon closer examination in each case, the enlarged vector field (spin) representation reveals notable deviations in the shapes of individual skyrmions and antiskyrmions from their standard form. For example, see inset of **e** for skyrmion-antiskyrmion pair.

* n.kiselev@fz-juelich.de
† aknandy@niser.ac.in

[1] B. Dupé, G. Bihlmayer, M. Böttcher, S. Blügel, and S. Heinze. Engineering skyrmions in transition-metal multilayers for spintronics. *Nature Communications*, 7(1), June 2016.

[2] Stefan Heinze, Kirsten von Bergmann, Matthias Menzel, Jens Brede, André Kubetzka, Roland Wiesendanger, Gustav Bihlmayer, and Stefan Blğel. Spontaneous atomic-scale magnetic skyrmion lattice in two dimensions. *Nature Physics*, 7:713–718, 03 2011.

[3] Markus Hoffmann, Bernd Zimmermann, Gideon P. Müller, Daniel Schürhoff, Nikolai S. Kiselev, Christof Melcher, and Stefan Blügel. Antiskyrmions stabilized at interfaces by anisotropic dzyaloshinskii-moriya interactions. *Nature Communications*, 8(1), August 2017.

[4] Vladyslav M. Kuchkin and Nikolai S. Kiselev. Skyrmions and antiskyrmions in monoaxial chiral magnets. *Phys. Rev. B*, 108:054426, Aug 2023.

[5] A. O. Leonov and M. Mostovoy. Multiply periodic states and isolated skyrmions in an anisotropic frustrated magnet. *Nat. Commun.*, 6:8275, 2015.

[6] Ashis Kumar Nandy, Nikolai S. Kiselev, and Stefan Blügel. Interlayer exchange coupling: A general scheme turning chiral magnets into magnetic multilayers carrying atomic-scale skyrmions. *Phys. Rev. Lett.*, 116:177202, Apr 2016.

[7] Filipp N Rybakov, Aleksandr B Borisov, Stefan Blügel, and Nikolai S Kiselev. New spiral state and skyrmion lattice in 3d model of chiral magnets. *New Journal of Physics*, 18(4):045002, apr 2016.

[8] Filipp N. Rybakov, Nikolai S. Kiselev, Aleksandr B. Borisov, Lukas Döring, Christof Melcher, and Stefan Blügel. Magnetic hopfions in solids. *APL Materials*, 10(11):111113, November 2022.

[9] P. J. van Laarhoven and E. H. Aarts. Simulated annealing: Theory and applications, 1987.
